# The nucleosome landscape of *Plasmodium falciparum* reveals chromatin architecture and dynamics of regulatory sequences

**DOI:** 10.1093/nar/gkv1214

**Published:** 2015-11-17

**Authors:** Philip Reiner Kensche, Wieteke Anna Maria Hoeijmakers, Christa Geeke Toenhake, Maaike Bras, Lia Chappell, Matthew Berriman, Richárd Bártfai

**Affiliations:** 1 Department of Molecular Biology, Radboud University, 6525GA Nijmegen, The Netherlands; 2 Parasite Genomics Group, Wellcome Trust Sanger Institute, CB10 1SA Hinxton, UK

## Abstract

In eukaryotes, the chromatin architecture has a pivotal role in regulating all DNA-associated processes and it is central to the control of gene expression. For *Plasmodium falciparum*, a causative agent of human malaria, the nucleosome positioning profile of regulatory regions deserves particular attention because of their extreme AT-content. With the aid of a highly controlled MNase-seq procedure we reveal how positioning of nucleosomes provides a structural and regulatory framework to the transcriptional unit by demarcating landmark sites (transcription/translation start and end sites). In addition, our analysis provides strong indications for the function of positioned nucleosomes in splice site recognition. Transcription start sites (TSSs) are bordered by a small nucleosome-depleted region, but lack the stereotypic downstream nucleosome arrays, highlighting a key difference in chromatin organization compared to model organisms. Furthermore, we observe transcription-coupled eviction of nucleosomes on strong TSSs during intraerythrocytic development and demonstrate that nucleosome positioning and dynamics can be predictive for the functionality of regulatory DNA elements. Collectively, the strong nucleosome positioning over splice sites and surrounding putative transcription factor binding sites highlights the regulatory capacity of the nucleosome landscape in this deadly human pathogen.

## INTRODUCTION

The genetic material of eukaryotic organisms is packed into chromatin, which has a profound impact on all DNA-associated processes, including transcription, replication, recombination and repair. The basic unit of the chromatin is the nucleosome, consisting of about 147 bp of DNA wrapped around a histone protein octamer. The nucleosomes are arranged into continuous arrays separated by small stretches of linker DNA. Since nucleosomes limit the access of DNA-binding proteins (DBP) to DNA, positioning and occupancy of nucleosomes within the chromatin fibre are key to the biological interpretation of the genome. Nucleosome occupancy refers to the proportion of cells in which a given region of DNA is occupied by a nucleosome, as well as the density of nucleosomes over this region (1). For example, transcription start sites (TSS) tend to contain a nucleosome-depleted region (NDR), enabling the formation of pre-initiation complexes, while inactive genes often reside in nucleosome dense chromatin (2). In contrast, nucleosome positioning refers to the degree to which the position of a nucleosome in relation to the DNA differs from random distribution. Further classification can be made regarding translational positioning (the exact DNA sequence occupied by the histone octamer) and rotational positioning (the orientation of the DNA helix towards the protein core in relation to the ∼10 bp helical turn of DNA) (1,3). For example, a nucleosome can reside on a preferred 147 bp location in relation to the DNA sequence (so-called ‘translationally well-positioned nucleosome’) and as such can inhibit binding of transcription factors to *cis*-regulatory elements present in this sequence or support transcription factor recruitment to the neighbouring linker DNA (1). Alternatively, some proteins can bind DNA also in a nucleosomal context—but only if their binding site is exposed on the nucleosome surface (‘preferred rotational positioning’ (1)).

The nucleosome landscape is defined and modulated by a delicate interplay between direct DNA-sequence-based and protein-mediated mechanisms (2). Intriguingly, many global features of the *in vivo* nucleosome landscape can also be observable in *in vitro* reconstituted chromatin, suggesting that the DNA sequence provides a basic blueprint for the nucleosome landscape (4). Indeed, 10 bp-periodicity of the AA/TT/AT/TA-dinucleotide has been found to create a favourable environment for nucleosome formation and is therefore predictive for positioning of nucleosomes (5–7). Other sequences (e.g. homo-polymeric nucleotide tracks) might be disfavoured for nucleosome formation and can lead to nucleosome depletion and positioning of neighbouring nucleosomes, simply by preventing their random sliding along the chromatin fibre (4,8). In addition to such intrinsic organization of the chromatin fibre, a wide variety of histone chaperones and chromatin remodelling enzyme complex(es) are known to modulate nucleosome occupancy, spacing and positioning (9). For example, the position and spacing of nucleosomes downstream of TSS are established by the activity of various remodelling enzymes in many eukaryotes (2).

The human malaria parasite, *Plasmodium falciparum*, is responsible for an estimated 584 000 deaths in 2013 alone (World Malaria Report 2013). Mounting evidence suggests that epigenetic regulatory mechanisms are critical for parasite survival and could serve as potential antimalarial drug targets (reviewed in (10)). In particular, the silencing and mutually exclusive expression of genes in the heterochromatic domain controls processes such as antigenic variation, commitment to gametocytogenesis and expression of alternative solute transporters enabling the parasite to survive in various hostile environments (10,11). These heterochromatic domains are demarcated by tri-methylation of lysine 9 at histone H3 (H3K9me3) (12,13) and consequent binding of heterochromatic protein 1 (HP1) (14,15). In contrast, regulatory regions within the euchromatic—but not heterochromatic—domain are delineated by a parasite-specific, double-variant nucleosome subtype, consisting of histone variants H2A.Z and H2B.Z (16–18). ‘Active’ post-translational modifications (e.g. H3K4me3, H3K9ac and H4K8ac) are primarily placed on these double-variant nucleosomes where the level of their incorporation correlates with promoter strength/gene expression (13,16,19–20). Although the epigenetic profile of these AT-rich euchromatic intergenic regions has been extensively investigated, we still lack sufficient insight on the nucleosome occupancy and positioning landscape of these important regulatory sequences.

The genome of *P. falciparum* is extraordinary in that it harbours the highest proportion of adenine and thymine bases of all genomes sequenced to date. The average genome-wide AT-content is 81%, often reaching 90–95% in intergenic regions; this is greater than that of those described for *Dictyostelium discoideum* and *Tetrahymena thermophila*, which have an average AT-content of 78%. Such extreme base composition challenges existing concepts on chromatin organization, which would predict that highly AT-rich sequences form rigid structures and are therefore refractory to nucleosome formation. However, the chromatin landscape of *Dictyostelium*, despite its high AT-content, was found to largely reflect the nucleosome structure of multicellular organisms (21). Similarly, we did not observe significant differential nucleosome occupancy between highly AT-rich intergenic and somewhat GC-richer coding regions after sequencing native MNase-digested *P. falciparum* chromatin (Supplementary Figure S3 in (16,17)) with an optimized protocol for Illumina sequencing (22). In contrast, significant reduction of nucleosome occupancy at intergenic sequences has been reported by other studies aiming to analyse the nucleosome landscape of *P. falciparum* (20,23–24). To resolve this discrepancy we extensively analysed the effect of various experimental steps on the MNase-seq profiles. Our analysis shows that nucleosomal signal is highly susceptible to depletion in AT-rich sequences due to over-digestion of chromatin, inefficient cross-linking of nucleosomes and/or unequal amplification of various DNA fragments. Importantly, the effect of these technical artefacts can exceed the relatively subtle signal resulting from the positioning of nucleosomes. Accordingly, many of the earlier findings, in particular the reported lower nucleosome occupancy in intergenic regions, (20,23–24) could be influenced or might even be the sole consequence of AT-dependent biases (see Supplementary Results and Discussion for details).

Therefore, we extensively optimized the MNase-seq protocol to meet the challenges of the *P. falciparum* genome. With this optimized protocol, we generated high-resolution nucleosome positioning profiles at eight stages of the *P. falciparum* intraerythrocytic development cycle. We observed clear positioning of nucleosomes in regulatory regions and around transcriptional landmark sites (e.g. TSS, ATG, splice donor and acceptor sites, STOP, TTS). Intriguingly, we find dynamic, local depletion of nucleosome occupancy on active TSS and reveal positioning of nucleosomes around ApiAP2 transcription factor binding sites. In combination with matched, strand-specific RNA-seq data, our MNase-seq profiles portray the static and dynamic components of the nucleosome landscape of *P. falciparum* regulatory DNA sequences.

## MATERIALS AND METHODS

### Parasite culture and gDNA extraction

*Var2csa*-selected 3D7 *P. falciparum* blood-stage parasites were cultured under standard conditions and synchronized as in (16). Parasite staging is comparable to Supplementary Figure S1B of (16) (Supplementary Table S4). Genomic DNA was extracted from synchronized ring stages as in (16) with some modifications (see Supplementary Materials and Methods for details).

### MNase-(ChIP)-Seq and controls

A 15 min formaldehyde cross-linking was performed and the optimal MNase + Exonuclease III digestion time was determined for each stage (for details see Supplementary Materials and Methods). Nuclei were mildly sonicated to free cross-linked nucleosomes from nuclear membranes. Soluble chromatin was collected and de-cross-linked directly (MNase-Seq) or used for chromatin immunoprecipitation (MNase-ChIP-Seq, anti-H4 core antibody), whereas insoluble debris was de-cross-linked as pellet control (see Supplementary Materials and Methods for details). Since our linear amplification protocol (LADS, (22)) was difficult to adapt to the NextFlex adapters of Illumina sequencing we used an optimized KAPA protocol for library preparation with comparable performance (as long as proper controls are included to correct for the remaining polymerase chain reaction (PCR) bias). Importantly, no size-selection step was applied to the MNase-Seq and control libraries allowing assessment of the full range of DNA fragments resulting from enzymatic chromatin digestion and *in silico* size-selection of paired-end sequenced libraries.

### Strand-specific RNA-seq

Total RNA isolation, oligo-dT-selection to enrich for polyA^+^ mRNA, RNA fragmentation and cDNA synthesis were performed as described elsewhere (25) with some modifications (see Supplementary Materials and Methods for details). To maintain directional information for strand-specific RNA-seq, dTTP was replaced by dUTP during second strand synthesis, followed by specific degradation of the ‘U’-base containing strand after adapter ligation.

### High-throughput sequencing and data analysis

MNase-Seq, gDNA, T40A α-histone H4 ChIP and T15 pellet control samples were sequenced 100 bp paired-end and strand-specific RNA-seq was sequenced for 92 cycles single-end on a HiSeq 2000 system (Illumina). Data processing and analysis was done with BioPerl (Version 1.6.9) (26), BioRuby (Version 1.4.3.0001) (27), rtracklayer (Version 1.20.4) (28), samtools (Version 0.1.19) (29), bedtools (Version 2.20.1) (30), Picard (Version 1.101) (31). Paired-end reads were clipped to 72 bp and all data was mapped with BWA sample (Version 0.6.2-r126). As a means to control for amplification and sequencing biases we sequenced a sonicated genomic DNA sample, which unavoidably has a different distribution of fragment lengths than MNase-digested and nucleosome-protected DNA. To avoid potential biases associated with such differences in the insert-size distributions (ISD), we transformed the ISD of the gDNA control using a sampling approach (for details see Supplementary Materials and Methods, Supplementary Figure S1F)). Polyadenylation sites from Siegel *et al*. (32) were used to call transcription end sites (TES). The most prominent TSS for about half of the *P. falciparum* genes was identified from full-length mRNA using template-switching oligo's and cDNA-sequencing (see Supplementary Materials and Methods). To identify sites displaying local dynamics in nucleosome occupancy we applied a minimal coverage filter and used an auto-correlation approach to reduce noise (for details see Supplementary Materials and Methods). (Positioned) nucleosomes were called using the DANPOS software (Version 2.2.1; dpos, paired = 1, nor = F) (33).

### DNA-pulldown

Native, mixed-stage *P. falciparum* nuclei were collected as in (13) with modifications (see Supplementary Materials and Methods, Supplementary Table S3). Native nuclear extract preparation was modified from (34), DNA pulldown, protein reduction, alkylation and digestion with Trypsin/LysC was performed as in (35) with modifications. Peptides were chemically labelled using the dimethyl-labelling approach (36) and sample-pools were purified on stage-tips as described previously (37). DNA pulldowns were performed in duplicate on the same nuclear extract using label-swap conditions and analysed on a QExactive mass spectrometer (Thermo Fisher Scientific) as in (35). Raw MS spectra were analysed by MaxQuant (version 1.4.1.2) (38) and downstream analysis was performed using the Perseus software package (version 1.4.0.20). Data was plotted in R and significant outliers (FDR0.05) were labelled.

### Data deposition

The Gene Expression Omnibus accession number for the MNase-seq, associated controls and strand-specific RNA-seq data reported in this paper is GSE66185. This data is also available at PlasmoDB (www.plasmoDB.org). The TSS data is available via European Nucleotide Archive (ERP010191).

## RESULTS

### High-accuracy MNase-seq reveals variably sized MNase-protected footprints at distinct genomic regions

**Figure 1.**
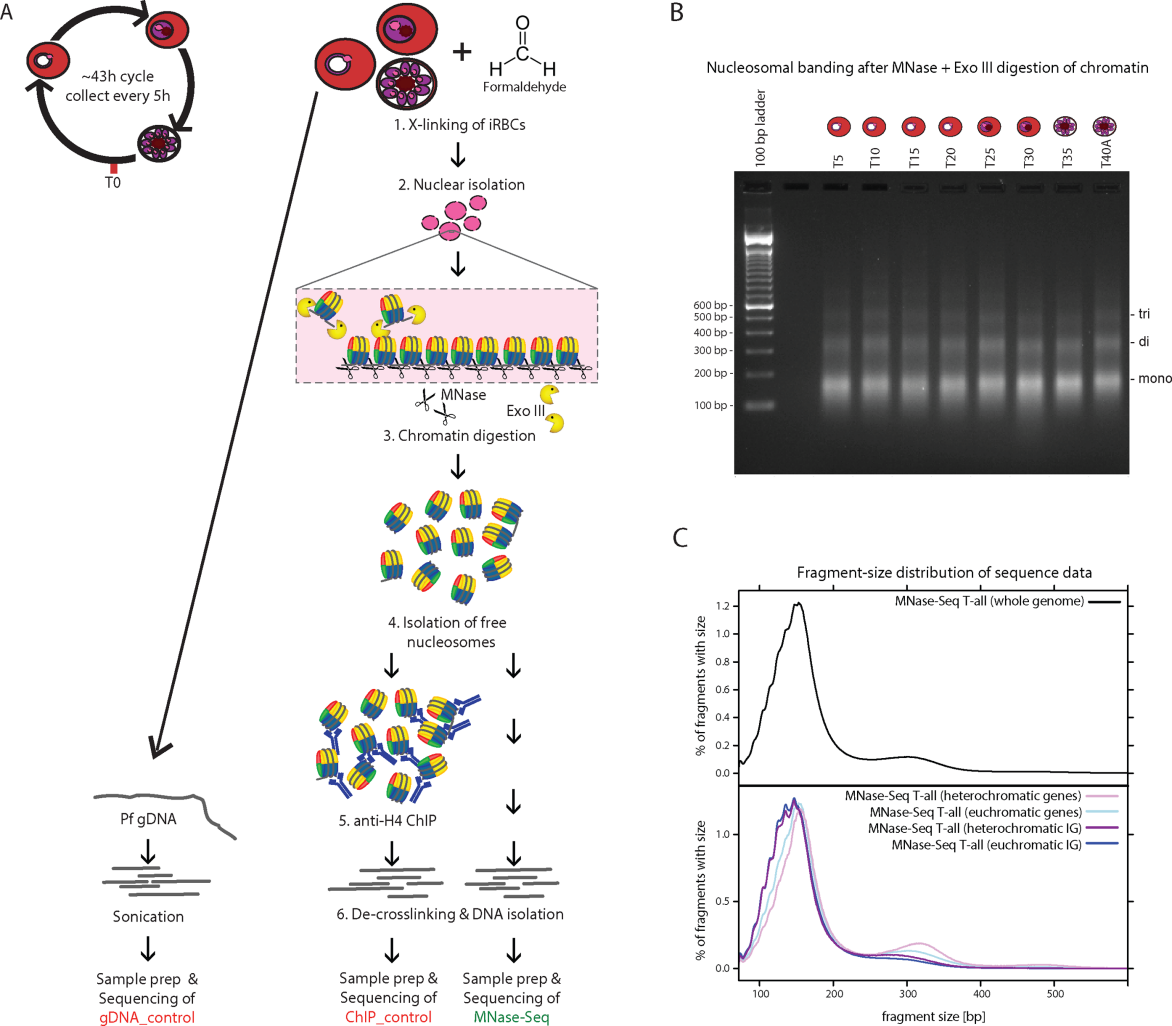
Highly controlled MNase-seq reveals variable size-distribution of nucleosomal DNA at distinct genomic regions. (**A**) Schematic of experimental procedure. For details on experimental procedures and controls see Supplementary Results and Discussion. (**B**) MNase + exonuclease III-digested chromatin separated on 2% agarose gel demonstrating comparable digestion across the stages (5–40 h post invasion, T40A is one of the technical replicates from 40 hpi). (**C**) Distribution of fragment sizes inferred from the uniquely aligned read pairs in the pooled MNase-seq libraries (T-all, top panel) and from MNase-seq T-all fragments aligning to hetero- (purple) or euchromatic (blue) genic (light) or intergenic (dark) regions (bottom panel), respectively.

To prevent possible artefacts and biases related to the extreme AT-richness, in particular due to amplifiability, digestability and formaldehyde-crosslinking efficiency of AT-rich sequences, we extensively optimized the MNase-seq procedure (Figure 1A; for details see Supplementary Results and Discussion). Synchronized *P. falciparum* 3D7 parasites were collected at eight different stages of intraerythrocytic development with 5 h intervals (T5-T40). After formaldehyde-crosslinking, chromatin was digested by a combination of micrococcal nuclease (MNase) and exonuclease III in order to reduce enzyme-mediated digestion-bias (39). We optimized the digestion conditions to ensure comparable fragmentation of chromatin obtained from different time-points producing mainly mono- and di-nucleosomal fragments (Figure 1B, Supplementary Figure S1A) and prepared sequencing libraries without selection of ‘mono-nucleosomal’ fragments. Sonicated genomic DNA was used as control to correct for biases introduced during library preparation and sequencing. MNase-seq libraries were subjected to paired-end Illumina sequencing (Supplementary Table S1) and fragment sizes were inferred from the distance between the genomic positions to which the 72 bp read pairs were uniquely mapped.

The size distribution of the fragments in the sequencing libraries (Figure 1C upper panel) was similar to that observed on agarose gel (Figure 1B) and comparable between stages (Supplementary Figure S1D). Notably however, di- and in particular tri-nucleosomal fragments were slightly under-represented in these libraries owing to the less efficient amplification of longer DNA fragments. After separating reads based on the genomic region which they originate from (i.e. eu- or hetero-chromatin, coding or intergenic) we observed clear differences in fragment size distributions (Figure 1C lower panel). As expected for more compacted chromatin, we observed the highest proportion of di-nucleosomal fragments in heterochromatic coding regions. Strikingly, the MNase-protected footprints in intergenic regions were substantially shorter than in coding sequences regardless whether they originated from hetero- or euchromatin (Figure 1C lower panel). This is unlikely the consequence of variant histone incorporation as double-variant H2A.Z/H2B.Z nucleosomes are found in intergenic euchromatic, but not heterochromatic regions (16–18). However, hetero- and euchromatic intergenic regions have comparable AT-content (86 and 87% respectively), which is higher than in the coding sequences (70% in hetero- and 78% in euchromatin). Therefore, the smaller size of the MNase-protected footprint in intergenic regions is most likely caused by the preferential digestion of AT-rich nucleosomes, instead of reflecting a truly smaller size of intergenic nucleosomes. Such ‘over-digestion’ could be the consequence of weaker association of AT-rich sequences to the histone octamer and/or result from the inherent cutting preference of the MNase enzyme at AT-rich sequences. Over-digestion preferentially occurs in helical DNA turn intervals on nucleosome-bound DNA (40), and this is indeed supported in our data by the ∼10 bp stepwise decrease of fragment sizes obtained from intergenic regions (Figure 1C). In conclusion, AT-richness confers intergenic regions sensitive to MNase ‘over-digestion’, thereby hampering their analysis with standard MNase-seq. The highly controlled digestion conditions and preservation of all sizes of nucleosomal fragments in our optimized MNase-seq procedure enabled accurate investigation of the nucleosome landscape of these important regulatory sequences.

### *Plasmodium falciparum* nucleosomes display a sequence-driven rotational setting and demonstrate translational positioning primarily in 5′ regulatory regions

**Figure 2.**
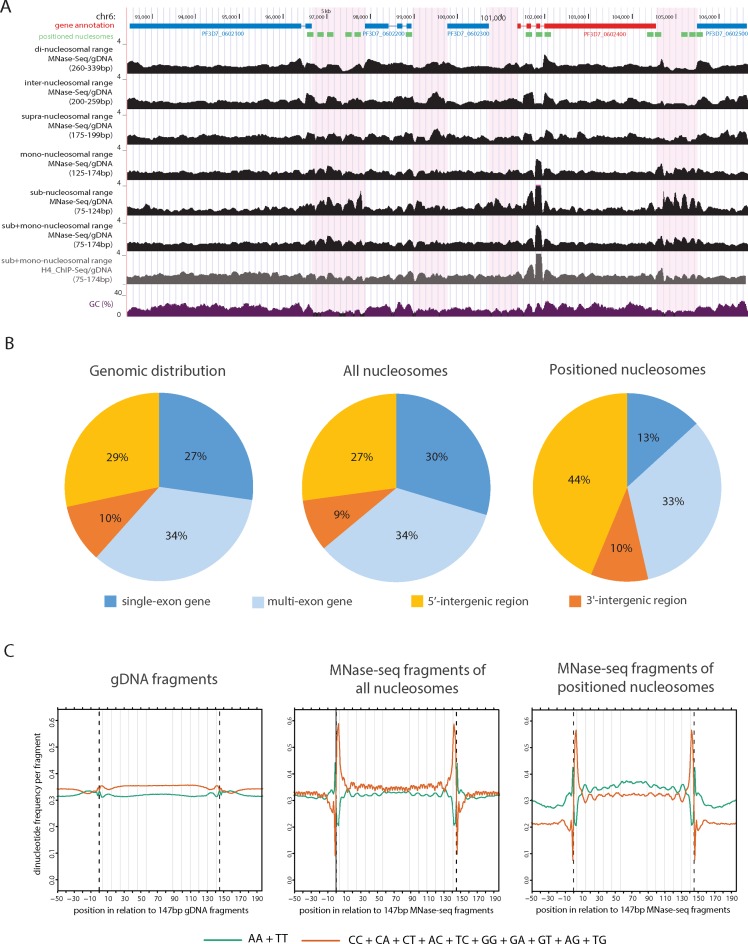
Well-positioned nucleosomes primarily localize to regulatory regions and display sequence-driven rotational positioning. (**A**) Screenshot displaying coverage plots of sub + mono-, sub-, mono-, supra-, inter- and di-nucleosomal range MNase-seq fragments as well as sub + mono range H4-ChIP-seq fragments corrected by matching gDNA control. Region shown is chr6:92 500–106 500. Blue gene: forward strand; Red gene: reverse strand; Green blocks: 10 000 most positioned nucleosomes as identified by DANPOS (**B**) Pie charts displaying the distribution of gDNA (left), all nucleosomes identified by DANPOS (middle) or the 10 000 most positioned nucleosomes (right) in various genomic regions (single-exon genes, multi-exon genes, 5′ intergenic regions, 3′ intergenic regions), respectively (**C**) Dinucleotide frequency profiles in and around 147 bp gDNA fragments (left), or MNase-seq fragments overlapping all (middle) or the 10 000 most positioned (right) nucleosomes called by DANPOS. Note: this analysis was performed exclusively on 147 bp long fragments selected based on paired-end mapping. Grey vertical lines are positioned in 10.5 bp intervals starting 4 bp inwards from the fragment ends. Note: the tri-nucleotide frequency observable in the middle panel is likely caused by codon bias in nucleosomes originating from coding sequences.

The above observations prompted us to further investigate the spatial distribution of MNase-seq fragments of different sizes. We grouped fragments into sub- (75–124 bp), mono- (125–174 bp), supra- (175–199 bp), inter- (200–259 bp) and di-nucleosomal (260–339 bp) size-classes and generated genome-wide coverage plots corrected using insert-size-matched gDNA controls (Figure 2A, Supplementary Figure S2A for raw data). Besides the relative depletion of di-, inter- and supra-nucleosomal fragments in intergenic regions (also observed on Figure 1C), no apparent pattern could be observed in the distribution of these fragment size classes in comparison to the gDNA control. In contrast, mono- and in particular sub-nucleosomal fragments showed clear enrichment over gDNA control mainly in intergenic regions as well as close to exon–intron boundaries and displayed peak patterns most likely corresponding to positioned nucleosomes (Figure 2A). Moreover, we observed stretches of nucleosome-sized ‘squared peaks’ in the sub- and mono-nucleosomal tracks showcasing well-positioned nucleosome clusters close to the ends of nearly all chromosomes (Supplementary Figure S2B). Importantly, the sub-nucleosomal fragments give rise to similar patterns in the histone H4 ChIP-control (Figure 2, Supplementary Figures S2A and S3A) or H3 ChIP (data not shown) verifying that the majority of these fragments indeed originate from nucleosomal DNA rather than DNA protected by other proteins (as in (41)). Accordingly, we conclude that the sub- and mono-nucleosomal size-classes are most informative for defining translational nucleosome positioning and therefore we used the combined sub + mono-nucleosomal fragments (75–174 bp) for all further analyses.

To further investigate the localization of positioned nucleosomes we use the software DANPOS (33) to define the most likely position of nucleosomes (‘all nucleosomes’) and select the 10 000 regions where the MNase-seq pattern indicated the highest level of positioning (‘positioned nucleosomes’, Figure 2A). Next, we calculated the proportion of these regions in 5′ and 3′ intergenic regions as well as in coding regions with or without introns (Figure 2B) and compared it to a random genomic distribution. This analysis confirmed our visual observation that positioned nucleosomes preferentially localized in 5′ intergenic regions. Furthermore, there is a relative enrichment of positioned nucleosomes in multi-exon genes (presumably on exon–intron boundaries) as compared to intronless genes.

To gain insight into potential sequence-directed positioning of nucleosomes we computed the AA/TT-dinucleotide frequency in and around all 147 bp MNase-seq fragments (Figure 2C). AA/TT-dinucleotide periodicity is predicted to reduce the energy required to bend DNA, resulting in sequences thermodynamically favourable for nucleosome formation and has been shown to dictate rotational positioning of nucleosomes in other organisms (4). Weak periodicity of these dinucleotides has also been reported earlier in *P. falciparum* MNase digested DNA in coding, but not in intergenic sequences (23). We observed a moderate, but unmistakable periodic pattern of AA/TT-dinucleotides at 10 bp intervals in MNase-seq fragments regardless whether they originated from positioned or all nucleosomes (Figure 2C), but not in gDNA fragments (Figure 2C). This suggests that AA/TT periodicity is a general feature of all *P. falciparum* nucleosomes regardless of their genomic origin or level of positioning. Interestingly, the combined frequency of all dinucleotides containing either cytosine or guanine showed an anti-phasic periodicity (i.e. 10 bp periodic pattern with a 5 bp shift) (Figure 2C). This might represent an independent signal for rotational positioning or simply be the consequence of the AA/TT dinucleotide periodicity in an extremely AT-rich genome.

In conclusion, by analysing sub- and mono-nucleosomal MNase-seq fragments we detect translational positioning of nucleosomes primarily in 5′ regulatory regions and in multi-exon genes. Furthermore, we found that 10bp AA/TT-dinucleotide periodicity-driven rotational positioning is a common feature of all *P. falciparum* nucleosomes despite the extremely AT-richness of the genome.

### Positioned nucleosomes frame the transcriptional unit in a largely transcription-independent fashion

**Figure 3.**
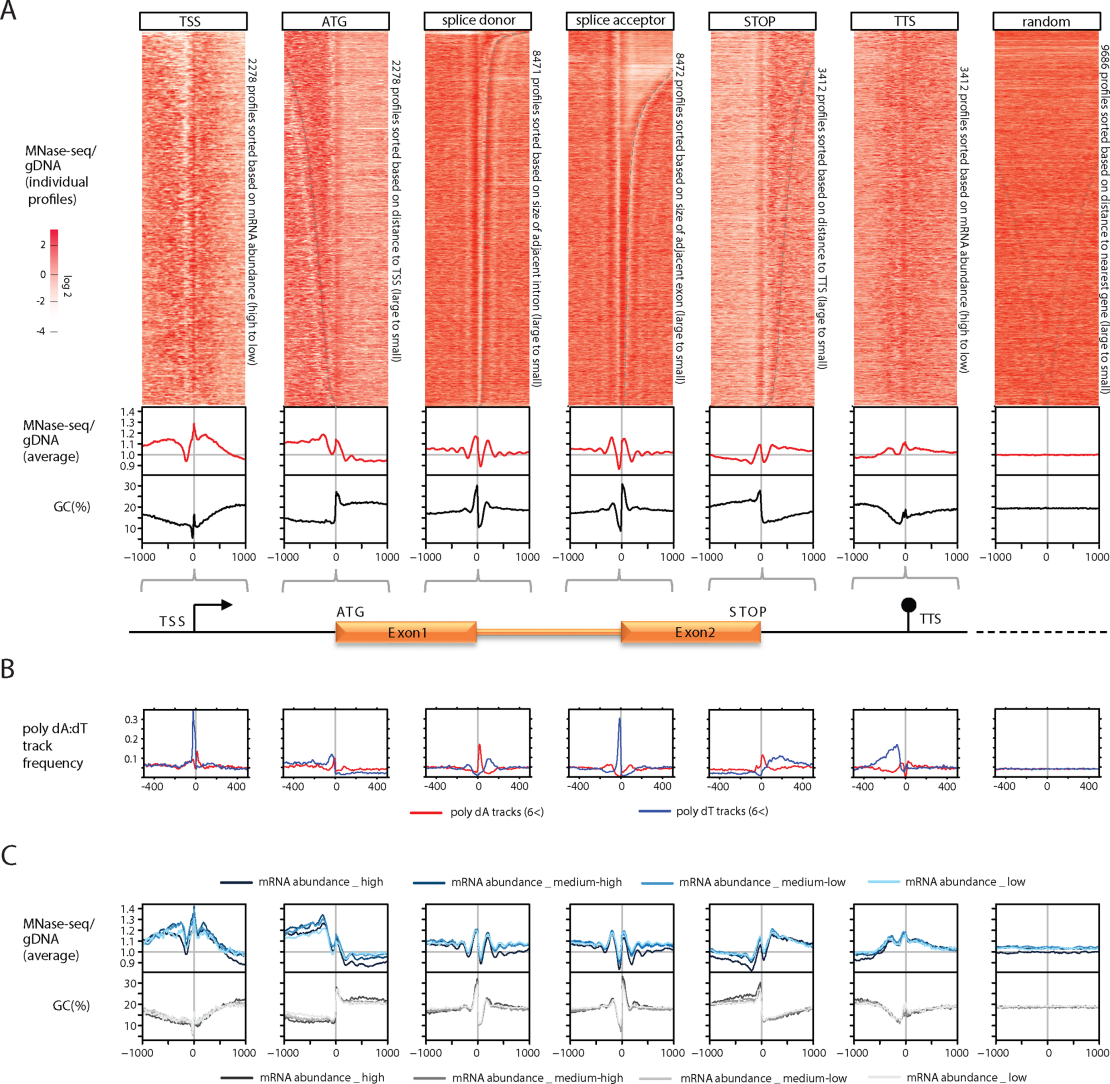
Positioning of nucleosomes on landmark transcriptional sites is largely transcription-independent. (**A**) Individual (heatmap, T40, log2-MNase-Seq/gDNA ratio) and average (line-graph, T-all) MNase-seq occupancy profiles around transcriptional landmark sites normalized by gDNA. The bottom panels show corresponding GC-content profiles. Ten-thousand non-overlapping random sites were selected uniformly across the genome as control. Note: for TSS and TTS individual occupancy profiles are sorted based on the mRNA abundance of the corresponding gene, while all other profiles are sorted based on distance to another landmark site (as indicated). Dashed grey line indicates the approximate position of the neighbouring landmark site. Heatmaps displaying individual profiles for all landmark sites sorted in three different ways (hierarchical clustering, mRNA abundance, distance to nearest landmark site) can be found in Supplementary Figure S4. (**B**) Frequency profiles of longer than 6 nt homopolymeric tracks (poly dA (red), poly dT (blue)) around transcription landmark sites. (**C**) Average normalized profiles (top) and GC-content profiles (bottom) for euchromatic genes in T40 separated into four different mRNA abundance quartiles. Darker colours reflect higher mRNA abundance.

In order to investigate the involvement of the nucleosome landscape in demarcating the transcriptional unit we visualized individual profiles of nucleosome occupancy around transcriptional landmark sites in heatmaps and plotted average profiles in line graphs (Figure 3A, top and middle panels). Importantly, given the dramatic difference in AT-content over these regions (Figure 3A bottom panel), the correction enabled by genomic DNA is essential to prevent incorrect conclusions about nucleosome positioning and occupancy (Supplementary Figure S3B, uncorrected profiles). After hierarchical clustering of individual nucleosome occupancy profiles (Supplementary Figure S4A), we observed positioning of nucleosomes upstream of the ATG, downstream of the STOP codon as well as overlaying ATG and STOP codon (Figure 3A, top panel, Supplementary Figure S4). Conserved placement of the first and last nucleosome within the coding region (also reported by (23)) is also evident from the average nucleosome occupancy profiles (Figure 3A, middle panel).

In *P. falciparum*, TSS were previously identified based on a full-length cDNA dataset (42). While this dataset has been instrumental in functional characterization of promoter sequences, due to its limited coverage, it does not support reliable genome-wide analysis of TSSs. Therefore, instead we used an RNA-seq dataset enriched for the 5′ end of transcripts (our unpublished data) to investigate the nucleosome landscape around the most prominent TSS of each gene. Analysis of 2278 start sites uncovered positioning of a +1 nucleosome right over the TSS in almost all analysed regions (Figure 3A). Additionally, nucleosome positioning upstream of the start site is evident from the hierarchically-clustered heatmap (Supplementary Figure S4A), but averaged out in the merged profile (Figure 3A, middle panel) due to a variable sized NDR separating them from the +1 nucleosome (Figure 3A and Supplementary Figure S4). In fact, these NDRs, similar to those in other organisms (2), appear to be a characteristic feature of the TSS in *P. falciparum* and likely organize the nucleosome landscape around this critical transcriptional landmark site. The presence of more than one TSSs is not uncommon in *P. falciparum* promoters (our unpublished data). Therefore, we also investigated the nucleosome landscape in 620 promoter regions, which contain at least two prominent TSSs (Supplementary Figure S5). The second TSS tends to have a similar, though less pronounced, chromatin organization as the primary TSS (a well-positioned nucleosome preceded by a NDR), which is most prominent when the distance between the two TSSs is about 200–300 bp. Therefore, promoter regions with structured NDR—nucleosome arrays might be more likely to give rise to multiple TSSs (Supplementary Figure S5).

When assessing the nucleosome landscape of transcription termination sites (TTS) (32), we again find that only the position of the nucleosome right over the TTS is conserved and therefore distinguishable in the average profile (Figure 3A, middle panel and Supplementary Figure S3). Similar positioning over TTSs has been observed in fission yeast, but with additional conservation of neighbouring nucleosomes. In contrast, nucleosome depletion has been reported surrounding the TTS of budding yeast (2) pointing towards species-specific differences in nucleosome placement surrounding TTS.

Interestingly, we observe the clearest positioning pattern around splice acceptor and donor sites, which often are enclosed by small arrays of positioned nucleosomes (Figure 3A). Such positioning of nucleosomes over splice sites has been observed in various other organisms (29) and has been implicated in guiding exon recognition by the splicing machinery.

Notably the *P. falciparum* genome is rather compact and therefore many of these landmark features localize closely to each other. To investigate the possibility that localization of nucleosomes at any given site is not an intrinsic feature of the region, but rather the consequence of nucleosome positioning at neighbouring sites we sorted the MNase-seq profiles based on the distance to the nearest landmark site (Figure 3A (ATG, splice donor, splice acceptor and STOP) and Supplementary Figure S4). This analysis suggests that the positioning of nucleosomes at individual sites is largely independent, but placement of these landmark sites about one nucleosome distance apart can lead to even stronger positioning (most apparent for splice donor and acceptor sites).

From the above analysis it is evident that the transcriptional unit is framed by well-positioned nucleosomes. This could result from the presence of specific sequences as has been reported for other organisms. Indeed we do observe local GC-content changes often accompanying the well-positioned nucleosomes at landmarks sites (Figure 3A, lower panel), which could potentially cause the observed positioning. Furthermore, combinations of polyA and/or polyT tracks were previously reported to influence local chromatin organization next to the ATG, STOP and TSS in both *Dictyostelium* and *Plasmodium* genomes (21,43). In addition, we observe enrichment of polyA/T tracks next to the splice junction and at the TTSs (Figure 3B) showing that they are commonly associated with all landmark features and hence likely contribute to nucleosome positioning at these sites.

Alternatively, the positioning of nucleosomes next to these landmark features could be a direct consequence of the transcription process. In order to investigate this possibility we generated matched strand-specific RNA-seq data and sorted individual profiles based on the steady state mRNA level of the corresponding gene (Figure 3A for TSS/TTS and Supplementary Figure S4B). We furthermore computed the nucleosome occupancy profiles for groups of euchromatic genes with low, medium-low, medium-high and high mRNA abundance (Figure 3C). Interestingly, the nucleosome occupancy profiles over most landmark sites appear to be largely comparable between genes with vastly different steady-state mRNA levels (Figure 3C). We detect the most pronounced differences between the transcription classes at regions surrounding the TSS (Figure 3A and C). In particular the depth and width of the NDR upstream of the TSS is more prominent for the group of highly transcribed genes. This suggests that the larger size of the NDR upstream of the TSS could either be the consequence or could potentiate higher levels of transcription.

In summary, we find largely transcription-independent and likely sequence-driven positioning of nucleosomes on/around key elements of the transcriptional unit. Furthermore, we detect an NDR immediately upstream of the TSS that is likely the site of RNA polymerase II pre-initiation complex formation.

### Dynamic occupancy of nucleosomes during intraerythrocytic development is most apparent around TSS

**Figure 4.**
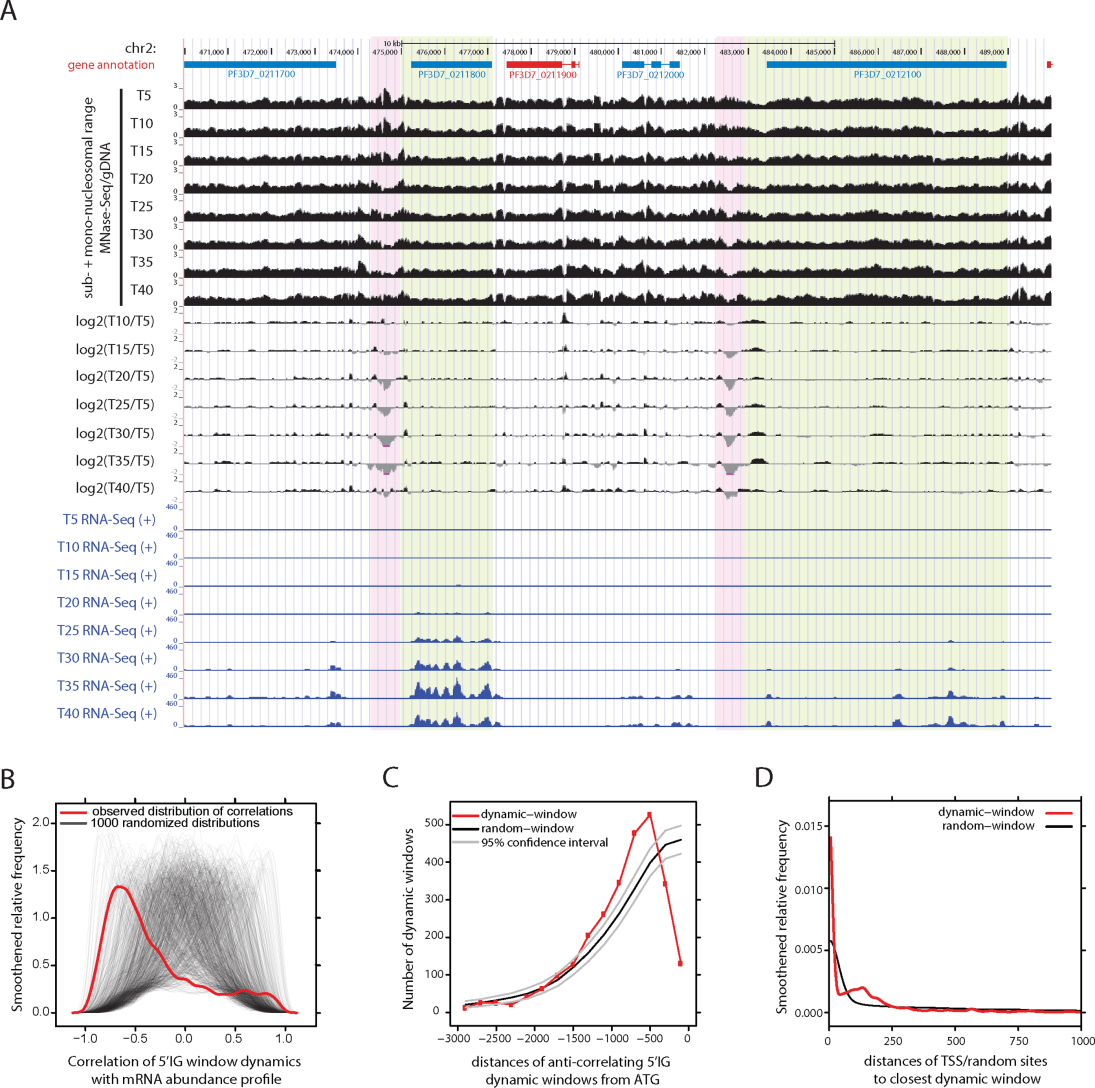
Dynamic nucleosome occupancy around transcriptional start sites during intraerythrocytic development. (**A**) Screenshot displaying absolute (gDNA-corrected MNase-seq coverage plots) or relative nucleosome occupancy (log2-ratio over T5) and steady-state mRNA expression (strand-specific RNA-seq). Region shown is chr2:470 000–490 000. Blue gene: forward strand; Red gene: reverse strand; Dynamic regions are highlighted pink, expressed downstream regions are highlighted green. (**B**) Observed (red) and 1000 randomized (transparent black) smoothed distributions (kernel density estimation) of Pearson correlations between the nucleosome-occupancy profile of upstream dynamically-occupied windows and the mRNA abundance profile of the downstream gene. Every black line shows the distribution of correlations for a single permutation of the time-point labels. (**C**) Observed (red) and expected (black, grey) distributions of distances between 3461 dynamically-occupied upstream windows (with anti-correlation between nucleosome-occupancy and mRNA abundance profile) and ATG of the downstream gene. Displayed are counts for 175 bp bins. The mean (black) and 95%-confidence intervals (grey) of the expected distribution were derived from 1000 randomizations of dynamic windows within the upstream regions. (**D**) Observed (red) and expected/randomized (black) smoothed distribution of distances of TSSs to the closest dynamic window.

The observation of a local NDR just upstream of the TSS that is most prominent for highly expressed genes (Figure 3A and B) could reflect an intrinsic feature of strong promoters, but could also stem from dynamic nucleosome loss upon activation of the gene. This prompted us to investigate the dynamics of the nucleosome landscape during intraerythrocytic development. Coverage plots obtained from eight developmental stages appear highly similar, however, local changes in nucleosome occupancy are highlighted by ratio-plots of the MNase-seq data from seven stages (T10–40) over the first time-point (T5, Figure 4A, Supplementary Figure S6). These changes in nucleosome occupancy could also be confirmed by qPCR. (Supplementary Figure S7). To investigate dynamic occupancy on a genomic scale, we identified 4821 75 bp-windows that displayed gradual changes in nucleosome occupancy levels (See ‘Materials and Methods’ section for details). The vast majority of these regions (∼80% of all dynamic windows) localized to euchromatic intergenic regions (33% of the genome). Furthermore, dynamic nucleosome occupancy is somewhat more likely to occur in 5′ compared to 3′ intergenic regions (2.5-fold enriched over expected). Such preferential localization towards 5′ intergenic regions suggests that dynamic nucleosome occupancy might occur at regulatory promoter elements.

To investigate this possibility, we determined the correlation between the nucleosome occupancy profiles of 5′ dynamic regions and the mRNA abundance profile of the downstream gene over eight time-points. For most windows nucleosome occupancy negatively correlated with steady-state mRNA level of the downstream gene (red line in Figure 4B). Importantly, this observation is independent of RNA-seq normalization methods; (16)) or statistical methods used (Supplementary Figure S8A–D). This indeed suggests that nucleosome depletion occurs at promoter elements upon transcriptional activity.

To further characterize these dynamically occupied regions, we determined their distance to the downstream gene. Importantly, nucleosome depletion upon transcriptional activity preferentially occurred ∼500–1000 bp upstream of the ATG (Figure 4C), while preferential localization was not observed for windows in which nucleosome occupancy positively correlated with mRNA abundance (Supplementary Figure S8E). Given this preferential localization we hypothesized that these NDR could overlay TSS. Therefore, we plotted for each TSS, the distance to the nearest dynamically occupied window and showed that nucleosome depletion in front of active genes preferentially occurs at or close to the TSS (Figure 4D). Notably, dynamically occupied 3′ IG regions did not exhibit correlation to mRNA levels or similar preferential localization in relation to the stop codon of the nearby gene (Supplementary Figure S8F–I). These findings collectively demonstrate that most prominent changes in nucleosome occupancy during intraerythrocytic development occur at TSS where nucleosome depletion is most evident when the downstream gene is active.

### Structured nucleosome landscape might discern functional from inert DNA motifs

**Figure 5.**
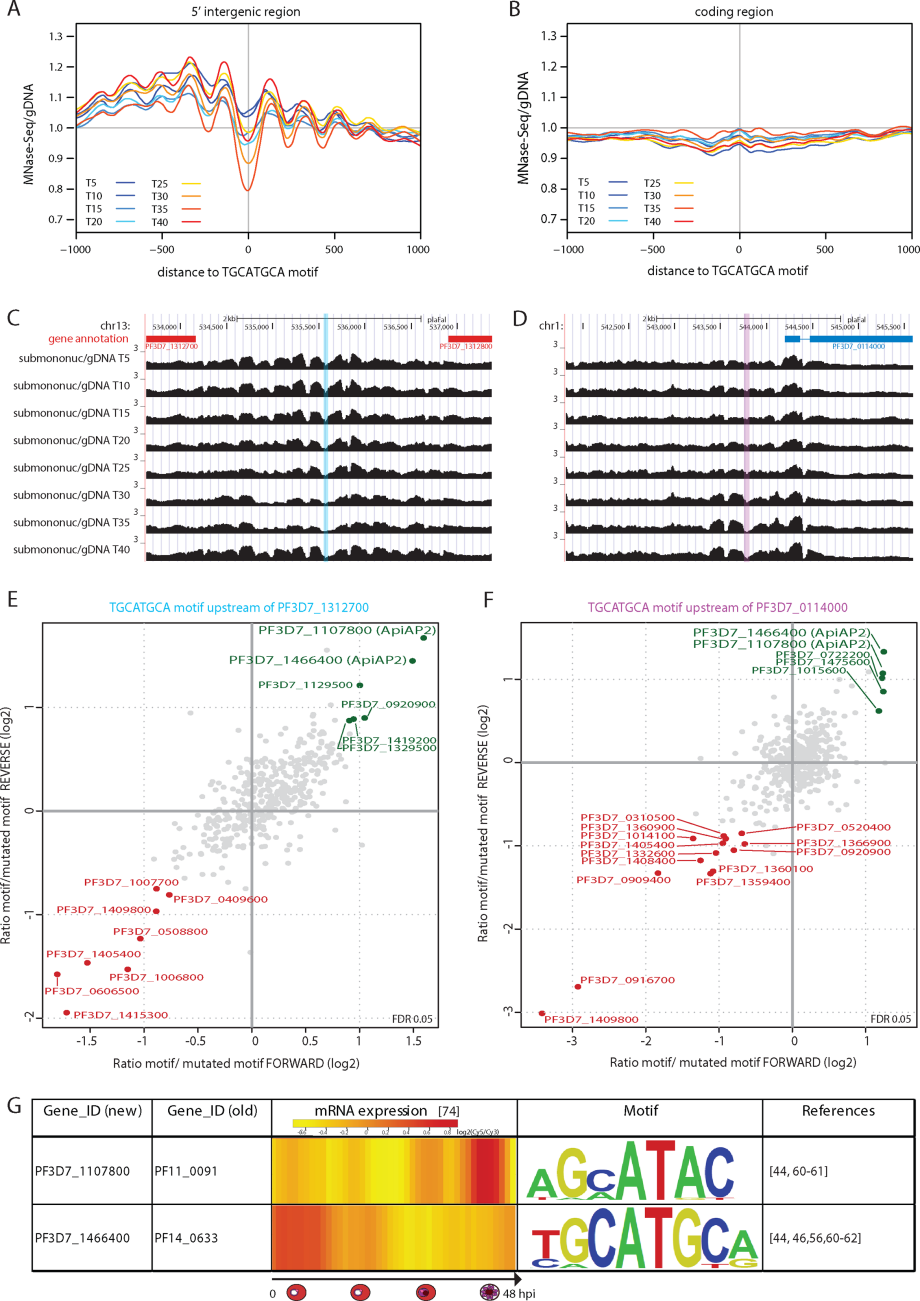
The nucleosome landscape can be predictive for functional DNA motifs. Smoothened, gDNA-corrected average nucleosome occupancy profiles around the top 394 or 726 scoring occurrences with *P*-value ≤ 8.43 × 10^−5^ of the TGCATGCA motif that are located in (**A**) upstream or (**B**) coding regions. (**C** and **D**) Screenshot of the nucleosome landscape (MNase-Seq-over-gDNA ratio for sub + mono-nucleosomal fragments) around two TGCATGCA motifs. Motif +/− 25 nt upstream of genes PF3D7_1312700 (C) and PF3D7_0114000 (D) is highlighted (blue and pink) and used for DNA pulldown experiments. Blue gene: forward strand; Red gene: reverse strand. (**E** and **F**) Scatterplot displaying the quantitative proteomic analysis of duplicate, label-swap DNA pulldowns performed with 500 μg mixed-stage nuclear extract. Statistically significant outliers (FDR < 5%) displaying preferential binding to TGCATGCA (green) or mutated-motif (red) containing probes upstream of genes PF3D7_1312700 (E) or PF3D7_0114000 (F) are marked. The y-axis of plot (F) is not auto-scaled and excludes a single data-point located at (x,y)-coordinates (−0.48, 5.06). (**G**) Table displaying steady-state mRNA level heatmaps (log2(Cy5/Cy3)-ratio from (59)) and the consensus-motif recognized by recombinant DNA-binding-domain for PF3D7_1107800 (from (44)) and PF3D7_1466400 (from (56)).

Given the delicate interplay between nucleosome positioning and binding of trans-acting factors, we decided to specifically investigate the nucleosome landscape and its dynamics around predicted binding sites of DBP. In absence of ChIP-seq data for experimentally-verified DBP-binding events during intraerythrocytic development, we made use of predictions for potential transcription factor (TF)-binding motifs. We selected three DNA motifs (GC-box, GTGCAC, TGCATGCA) for which multiple lines of evidence suggest that they might be bound by DBPs (44,45). Amongst these, the clearest nucleosome organization was observed around sequences with close-to-perfect matches to the TGCATGCA consensus motif in 5′ intergenic regions (Figure 5A), but not in coding sequences (Figure 5B). Interestingly, we observe most prominent patterning over TGCATGCA-sites at later stages of development (T30–35) (Figure 5A), which could be indicative for DBP binding. Strikingly however, the transcript of the ApiAP2-type transcription factor that is predicted to bind this element, PF3D7_1466400 is most abundant at the early stages of development ((44) and Figure 5G) and the *P. bergei* orthologue of this factor appears to be largely dispensable for blood stage development (46). Therefore, we wondered whether another factor might be binding to this motif in late stages of asexual RBC development. To resolve this discrepancy we selected two genomic regions, which contained a TGCATGCA motif and displayed marked nucleosome landscape dynamics during blood stage development (Figure 5C and D). Next we used ∼60 bp double-stranded DNA probes matching these regions to affinity purify proteins that bind to the TGCATGCA motif, but not mutated DNA elements and analysed them by quantitative proteomics (Figure 5E and F, Supplementary Table S2). For both regions, we find the earlier predicted (PF3D7_1466400 (44)) as well as one other ApiAP2-type transcription factor (PF3D7_1107800) preferentially binding to the motif-containing probe. Interestingly, the single AP2-domain of this second transcription factor has been shown to bind a similar sequence motif *in vitro*, and the corresponding mRNA is most abundant at the later stages of the blood stage development (Figure 5G). Consequently, it seems plausible that the binding of this second or both of the two transcription factors is responsible for the observed local changes of the nucleosome landscape around TGCATGCA motifs. These finding suggests that dynamic nucleosome occupancy can be indicative of a transcription factor binding event and aid the separation of functional from inert DNA motifs for DBP-elements within the *P. falciparum* genome.

## DISCUSSION

In this study we generated high-resolution nucleosome profiles of the *P. falciparum* genome at eight time-points of intraerythrocytic development. Our aims were two-fold: to understand how chromatin is organized on this AT-rich genome and to explore the relevance of the nucleosome landscape in guiding the transcriptional process. However, before we could reliably analyse the nucleosome landscape of *P. falciparum*, we had to overcome several methodological challenges. Given the intrinsic preference of MNase to cleave next to A or T nucleotides, preferential degradation of AT-rich nucleosomal DNA has been reported (39). Accordingly, excessive MNase digestion can result in degradation or even complete loss of AT-rich nucleosomes (40), while leaving GC-rich nucleosomal and also linker DNA undigested. We carefully titrated digestion times to avoid extensive over-digestion and ensure comparable digestion of chromatin from different parasite stages. Furthermore, we fragmented chromatin by combined MNase and exonuclease III digestion, which has previously been shown to reduce MNase-bias on artificial DNA sequences (39). Indeed, somewhat improved digestion of MNase-‘resistant’ GC-rich linkers in our MNase-seq data was observed upon addition of exonuclease III (data not shown). In addition to providing a challenge for even chromatin digestion, the AT-rich *P. falciparum* DNA is notoriously problematic for unbiased library preparation, amplification and sequencing (22,47–48). Consequently, a proper control to correct for biases introduced at these stages of the procedure is vital. We choose to use a sonicated gDNA control, which will correct for all sequencing-based artefacts. Indeed, our gDNA control rectifies the major part of the substantial anti-AT-bias observed in raw MNase-seq data (Figure 3B and Supplementary Figure S3). Nonetheless, a relative over-digestion of AT-rich intergenic nucleosomes is evident (Figure 1C), and therefore analysis of sub-nucleosomal DNA fragments was pertinent to obtain nucleosome positioning/occupancy information in regulatory regions. Moreover, we observed a fragment-length-dependent PCR-mediated AT-bias (Supplementary Figure S2A), uneven detainment of DNA sequences within the insoluble nuclear membrane pellet (T15 Pellet control, see Supplementary Results and Discussion for details) as well as a ChIP-mediated AT-bias likely caused by the less efficient cross-linking of AT-rich DNA (T40 ChIP control). As a consequence, we believe that a general caution needs to be applied when making statements on nucleosome occupancy differences between genomic regions and/or samples with different extents of chromatin digestion. For example, the reduction of intergenic nucleosomal signal to the extent as it has been claimed by the LeRoch laboratory (23,24) is not observed in our dataset after gDNA correction and is likely the result of technical impediments as described above (see Supplementary Results and Discussion for further details).

Nucleosome positioning in *P. falciparum* is centred around the transcriptional unit, with positioned nucleosomes being detected on the TSS, ATG, splice donor and acceptor site, STOP and TTS (positioning of the first and last nucleosome of coding sequences has also been reported by (23)). However, contrary to observations in other organisms, we discovered the strongest positioning signals over splice donor and acceptor sites (Figure 3A). Nucleosome positioning over splice junctions has been reported for evolutionary diverged eukaryotes (29,49). In *Caenorhabditis elegans* and human this positioning is mainly restricted to the exons (29). Similar to what we observe for *P. falciparum* (Figure 3B) the positioning is independent of gene expression (29) and consequently seems to be encoded in the DNA either directly or via recruitment of chromatin remodellers. In particular, distinctly higher GC-content of exonic sequences has been implicated to promote exon recognition in genes with long introns (50) in warm-blooded animals. Similarly, exons and introns of *P. falciparum* have distinctly different GC-content (average 23.5 and 13.5% respectively), but introns are on average only 180 bp in length. Accordingly, in case of very short introns nucleosomes are mainly positioned over the GC-richer exons leaving the introns largely nucleosome-free. While in case of longer introns an intronic nucleosome can ‘sit in the AT-richer pocket’ between two exons (Figure 3A). Both of these scenarios, together with the strong enrichment of polyA/T tracks around splice sites (Figure 3C), could explain strong positioning at exon–intron boundaries. Furthermore alternating short introns and exons (which are not uncommon to the *P. falciparum* genome) can give rise to arrays of positioned nucleosomes. Interestingly, it was recently elegantly demonstrated that the well-positioned +1 nucleosome in *D. melanogaster* slows down RNA Pol II progression (51). Consequently, it is plausible to hypothesize that well-positioned nucleosomes over splice sites also impose a stronger boundary to RNA Pol II progression and thereby promote co-transcriptional splicing events.

Since the currently available full-length cDNA dataset (42) only provides reliable TSS positions for a few hundred *P. falciparum* genes we made use of a 5′ end enriched RNA-seq dataset to map the most prominent start site for about half of the *P. falciparum* genes. Analyses of these sites substantially clarified the nucleosome organization around the TSS and revealed a NDR as its most prominent feature (Figure 3). However, even in this improved TSS landscape the positioning of the +1 as well as the +2 nucleosome is weak compared to stereotypic core promoter architecture of other eukaryotes (2). Although several theories might explain this observation, it could very well be possible that different classes of genes (e.g. housekeeping versus stage-specific genes, bi- versus uni-directional promoters) display a different nucleosome landscape, which results in a much fuzzier pattern when data from these genes are merged. An elegant recent study revealing distinct core promoter architectures for zygotic and embryonic genes in vertebrates (52) is in line with this hypothesis. While we have not yet identified functionally distinct gene classes with different core promoter architecture, we noticed that the width of the NDR varies between individual core promoters and genes with a higher level of transcription tend to have a wider NDR (Figure 3). Such a wider NDR might provide better access for the transcription initiation machinery and thereby could contribute to a higher transcriptional output. Alternatively, it was demonstrated that phasing of nucleosomes downstream of TSSs is achieved by the action of chromatin remodelling enzymes. Indeed, yeast lacking the ISW1/2 and CHD1 remodellers display a core promoter landscape more similar to the one we observed in *P. falciparum* (2). Therefore, it is possible that malaria parasites either lack some of these enzymes or do not utilize them to phase nucleosomes downstream of the TSS. Whatever, the exact reason is, it seems that the nucleosome architecture around TSS is less delicately defined in *P. falciparum* than in other eukaryotes. The mere presence of a NDR flanked by positioned nucleosomes, therefore appears to be sufficient information for transcription initiation.

Despite intense investigation in the nucleosome field, it is still controversial to which extend the genomic sequence influences the *in vivo* nucleosome landscape (3). In many organisms, a 10 bp-periodicity of AA/TT/AT/TA-dinucleotides when the minor groove faces inwards to the nucleosome has been described (3). Earlier analysis of MNase digested chromatin from *P. falciparum* suggested that AA/TT-dinucleotides periodicity is absent in AT-rich intergenic regions, while weak periodicity was observed at coding sequences (23). We however observe clear AA/TT-dinucleotides periodicity in all MNase digested fragments, showing that sequence-dictated rotational positioning is maintained even in case of extreme genomic AT-content. In addition to di-nucleotide frequencies, longer sequence motifs have been reported that either favour or disfavour nucleosome formation. For example, ATATA- and TATAT-type repeats were found under-represented in MNase digested chromatin fragments of yeast and *P. falciparum* (23,53). We also found these sequences markedly under-represented in all 147 bp MNase-seq fragments, but detected a similar sharp decrease in TATAT/ATATA-repeat frequency of 127 and 107 bp ‘over-digested’ MNase-seq fragments, while fragments originating from the gDNA control display a gradual reduction (Supplementary Figure S2C and D). Consequently, our controls suggest that the reduced frequency of ATATA/TATAT-repeat sequences is most likely the consequence of preferential cutting of such repeats by MNase in combination with depletion during library preparation and does not support the statement that these sequences are refractory to nucleosome formation. Long polymeric AAAAAA- or TTTTTT-stretches have also been claimed to possess nucleosome-repelling properties due to their intrinsic stiffness (2). A specific combination of polyA and polyT tracks has indeed been found to be a common feature of transcriptional or translational start and translational end sites in *P. falciparum* (43) and in *D. discoideum* (21). In our data, we also find clear enrichment of these sequences next to transcription start, splice and transcriptional termination sites. Interestingly, the enrichment of these sequences seem to show some level of correlation with the level of nucleosome positioning, suggesting that this association might be functionally relevant. A recent study by the Kornberg lab shows that lower occupancy of nucleosomes over polyA/T-tracks at *Saccharomyces cerevisiae* promoters is largely due to the activity of the chromatin remodeller RSC (54) with only a minor contribution of physical DNA properties. Accordingly, it is not unthinkable that in *P. falciparum* polyA/T homo-polymeric tracks are not nucleosome repelling by intrinsic properties, but might be employed to shape the nucleosome landscape of selected regions by specific remodelling activity. In line with this hypothesis, Polson and Blackman (55)) demonstrated that polyA/T tracks in the *P. falciparum* calmodulin gene promoter are specifically bound by a protein or complex, which could exhibit or recruit such remodeller activity.

The nucleosome landscapes obtained from differently staged asexual parasites are largely similar (Figure 4A), but display local depletion mainly 500–1000 bp upstream of genes that correlated with mRNA abundance of the downstream gene. These sites tend to overlay TSS and therefore we most likely detect eviction of nucleosomes from important regulatory elements prior to or as a consequence of transcriptional activation. In addition, we observe clear positioning and dynamics of nucleosomes around TGCATGCA motifs localized in intergenic, but not in coding regions. The TGCATGCA motif is one of the most over-represented motifs in apicomplexan genomes (56) and occurs upstream of over 1/3 of genes if moderate resemblance to the consensus sequence is allowed. We therefore stringently selected only close-to-perfect matches to the consensus sequence resulting in 394 upstream or 726 CDS localized motifs. To our surprise, while the depletion of nucleosomes is clearest at late stages of intraerythrocytic development (∼T35) it correlates neither to the steady-state RNA level of the DBP predicted to bind this motif (PF3D7_1466400, (56) and Figure 5G, highest expressed at ring stage), nor to the expression profiles of downstream genes (data not shown). This seeming contradiction could be explained in various ways: (i) the protein abundance and/or activity of PF3D7_1466400 differs from its steady-state RNA level, due to post-transcriptional and/or post-translational regulatory mechanisms; (ii) it is not or not only PF3D7_1466400 that binds this motif *in vivo*, (iii) the observed nucleosome dynamics are not the consequence of differential TF-binding, but of recruitment of chromatin remodelling complexes at later stages of development. To investigate the second possibility we performed DNA pulldown experiments using DNA probes resembling the genomic sequence of two 5′ intergenic region localized TGCATGCA motifs that display nucleosome landscape dynamics during asexual development. Indeed we identify a second AP2 protein, PF3D7_1107800, binding this motif in both regions. As this AP2s steady-state RNA abundance peaks around T35 (Figure 5G), it seems plausible that this transcription factor might be responsible for the observed structuring of the nucleosome landscape surrounding the TGCATGCA motif in the second half of the blood-stage cycle. In conclusion, our analysis of the TGCATGCA motif provides proof-of-principle on how nucleosome landscape-guided identification of TF-binding events might contribute to dissection of transcription-factor mediated regulation of gene expression in *P. falciparum*. An exciting area of research that awaits further investigation is to determine for how many other DNA motifs the nucleosome landscape will be able to distinguish between functional and inert elements.

All-in-all our adapted MNase-seq procedure allowed for the first time accurate characterization of the nucleosome landscape of the highly AT-rich regulatory regions in the *P. falciparum* genome. It revealed nucleosome positioning primarily on 5′ intergenic regions as well as splice donor and acceptor sites and the presence of a local NDR directly upstream of the TSS which is more pronounced for highly expressed genes. Dynamic, local nucleosome depletion during asexual blood-stage development is observed over TSS and correlates to temporal gene expression. Furthermore, the highly structured and dynamic nucleosome landscape over 5′ intergenic region—but not CDS—localized TGCATGCA motifs aided the identification of a second ApiAP2 transcription factor putatively binding this motif *in vivo*. In summary our study revealed how positioning of nucleosomes provides a structural and regulatory framework to the transcriptional process. Now, the challenge ahead lies in identification of parasite-specific DBPs, chaperones and/or chromatin remodellers that together shape this landscape and could function as potential anti-malarial drug targets.

## Supplementary Material

SUPPLEMENTARY DATA
