# Supplementary material for: The nucleosome landscape of Plasmodium falciparum reveals chromatin architecture and dynamics of regulatory sequences
Source: Nucleic Acids Res. 2015 Nov 17;44(5):2110–24. doi: 10.1093/nar/gkv1214 (PMC4797266; doi:10.1093/nar/gkv1214)
Supplement: SUPPLEMENTARY DATA [file supp_44_5_2110__index.html]

The nucleosome landscape of Plasmodium falciparum reveals chromatin architecture and dynamics of regulatory sequences — The nucleosome landscape of Plasmodium falciparum reveals chromatin architecture and dynamics of regulatory sequences — SUPPLEMENTARY DATA 

# The nucleosome landscape of *Plasmodium falciparum* reveals chromatin architecture and dynamics of regulatory sequences

## SUPPLEMENTARY DATA

- SUPPLEMENTARY DATA
- SUPPLEMENTARY DATA
- SUPPLEMENTARY DATA
- SUPPLEMENTARY DATA
