# Supplementary material for: The nucleosome landscape of Plasmodium falciparum reveals chromatin architecture and dynamics of regulatory sequences
Source: Nucleic Acids Res. 2015 Nov 17;44(5):2110–24. doi: 10.1093/nar/gkv1214 (PMC4797266; doi:10.1093/nar/gkv1214)
Supplement: SUPPLEMENTARY DATA [file supp_gkv1214_nar-02108-h-2015-File007.pdf]

The Nucleosome Landscape of *P. falciparum* Reveals Chromatin Architecture and Dynamics of Regulatory Sequences

Supplementary Results & Discussion:

Given that extensive description of all technical details would have obscured readability of the main text, but is critical to generation of high-quality nucleosome maps, we decided to dedicate a Supplementary section to these important observations. Here we also describe in detail in which way our results differ from earlier findings and discuss how technical biases might explain these discrepancies.

Formaldehyde crosslinking

Our previous MNase-digested native chromatin maps, which we used as a control for ChIP-seq data (1), did not reveal the squared shaped peaks typical of well-positioned nucleosomes in MNase-seq data. We hypothesized that exact positioning information might be lost due to nucleosome movement during MNase-digestion (which is performed at 37°C), as has been earlier suggested in (2). In order to prevent such movement, formaldehyde cross-linking was performed directly upon removal of parasite cultures from the 37°C incubator. Cross-linking indeed resulted in the appearance of square-shaped nucleosome peaks at plenteous genomic locations (Supplementary Figure 2), evidencing the existence of well-positioned nucleosomes in the *P. falciparum* epigenome and the superiority of cross-linked based approaches for analysis of exact nucleosome positioning. Formaldehyde cross-linking, on the other hand, also has some less beneficial effects that needed to be taken into consideration (see below).

Uneven retainment of chromatin in the nuclear pellet

Upon formaldehyde cross-linking not only DNA and histones become covalently linked, but chromatin can also be cross-linked to proteins of the nuclear skeleton. To efficiently extract all chromatin fragments, digestion of the chromatin was followed by mild sonication and soluble nucleosomes were isolated for MNase-Seq (see Figure 1A). Nonetheless, 7-16% of total chromatin remained associated with the nuclear skeleton (table in Supplementary Figure 1B). To investigate if any particular chromatin domains remain preferentially associated to the nuclear membrane or skeleton we de-cross-linked the nuclear pellet (called Pellet control, Figure 1A), isolated the DNA (Supplementary Figure 1) and sequenced a single pellet sample (T15, Supplementary Figure 1E) alongside our MNase-Seq libraries. T15 Pellet-Seq data displayed strong enrichment of centromeric regions and accordingly these regions are somewhat underrepresented in the corresponding MNase-seq profiles (data not shown). The cause for this strong enrichment of centromere DNA in the Pellet-Seq sample is currently unknown, but could potentially be a consequence of 3D genome organisation placing this DNA in close association to nuclear membranes. In line with this possibility we have earlier observed that centromeric regions do cluster and might localise to the nuclear periphery (3). Importantly, however, besides the enrichment of centromeric sequences preferential retainment of other genomic regions in the nuclear pellet was not apparent.

MNase-seq versus MNase-ChIP-seq

Nucleosomal maps can be generated from MNase-digested chromatin directly, or upon chromatin immunoprecipitation (ChIP) using an anti-histone antibody. Histone H3 or H4 ChIP is commonly applied to ensure that only DNA fragments protected by nucleosomes are retained. We prepared our nucleosome maps both ways to test which procedure – if any – works better. Soluble chromatin collected after brief sonication of the nuclei, was either directly used for de-cross-linking and sequencing library preparation (MNase-Seq libraries, all 8 stages) or first subjected to an anti-histone H4-core histone ChIP followed by de-cross-linking and sequencing library preparation (ChIP-Seq control sample, T40 stage only). Although the overall pattern of MNase-Seq and ChIP-seq datasets was very comparable (similar shaped peaks were found at identical positions) to our surprise we observed a consistent, ~1.25-1.5-fold reduction in intergenic (Supplementary Figure 2) and a ~3-fold reduction of centromeric signal (data not shown) in the ChIP-seq library specifically. We confirmed intergenic and centromeric depletion by ChIP-seq and ChIP-qPCR on mixed stage parasites using both histone H3-core (Abcam Ab1791, lot GR88948-1, data not shown) and histone H4-core antibodies (data not shown). As the observed depletion correlated almost perfectly with genomic AT-content we decided to further investigate whether this represents truly reduced nucleosome occupancy at intergenic regions or could be a result of technical artefacts that are prone to occur on these high AT-regions. We reasoned that protection of MNase-digestion by non-nucleosomal protein complexes (remodellers, transcription factors, etc.) would not be expected to yield nucleosomes-sized fragments/ladders and therefore should result in increased levels of non-nucleosome-sized fragments. However, upon comparison of the insert-size distribution of our paired-end sequenced MNase-Seq and ChIP-Seq libraries we did not see an increase of inter- or other non-nucleosome-sized fragments (Supplementary Figure 1E). Furthermore, if there would be substantial background of non-nucleosomal signal in the MNase-Seq libraries, this should result in additional peaks appearing in the genome-browser tracks, which we also did not observe. On the other hand, cross-linking is rather inefficient (2) and the high AT-content of intergenic regions could further reduce cross-linking efficiency as formaldehyde protein-DNA crosslinks are primarily formed between the bases adenine, cytosine and guanine and the amino-groups of amino-acids lysine and arginine, while thymidine is not or only poorly cross-linked (4,5). As a consequence, incomplete cross-linking would primarily affect the AT-rich intergenic and centromeric regions and non-cross-linked DNA would be lost during washing steps in the ChIP procedure. Of note, differential nucleosome composition (e.g. incorporation of histone variants) could also possibly result in differential cross-linking efficiency. However, since loss in intergenic signal is observed both for euchromatic intergenic regions (containing double-variant H2A.Z/H2B.Z nucleosomes) and heterochromatic intergenic regions (containing canonical H2A/H2B nucleosomes) this is unlikely to explain the observed effect. Thus, although we cannot formally exclude that biological causes might underlie the observed difference between our MNase-Seq and MNase-ChIP-seq datasets, we believe these differences are likely caused by an artificial loss of AT-rich fragments during the ChIP procedure. Therefore, we decided to use MNase-Seq libraries, which appear to be more reliably reflecting nucleosome occupancy.

Sequence preference of MNase

MNase has an intrinsic preference to cleave adenine or thymine over guanine or cytosine nucleotides, both for its endonuclease as well as exonuclease activities (6,7), which results in an unavoidable bias in MNase-Seq data. The extent to which this bias influences MNase-mediated nucleosome maps is subject to fierce debates in the field (6,7). Two recent studies reporting dissection of the *S. cerevisiae* (8) and *S. pombe* (9) nucleosomal landscape at single-base resolution using a chemical mapping approach settled the argument by uncovering that MNase-generated nucleosome maps - although somewhat noisy - provide an accurate reflection of the 'true' nucleosome landscape. Nonetheless, caution is required when applying MNase, especially on a genome where AT-content is not evenly distributed over different genomic regions (*Pf* intergenic ~86% AT, euchromatic coding ~78% AT, heterochromatic coding ~70% AT). In order to reduce MNase-directed bias we used a combined MNase and exonuclease III treatment. This has previously been shown to reduce MNase-bias (7) and indeed improvement digestion of MNase-resistant poly dG/dC tracks at the ends of MNase digested fragments also in our dataset (data not shown).

However, AT-rich intergenic sequences (Figure 1C) and in particular AT-tracks (Supplementary Figure 2C) are prone to over-digestion (8). Therefore, we decided to slightly under-digest our chromatin, given that digestion to solely mono-nucleosomes was impossible to achieve without heavily over-digesting the sample, which could potentially result in loss of or inaccurate positioning of nucleosomes. Careful titration of digestion times resulted in very comparable digestion rates for the different samples (Figure 1B) and enabled better comparability of nucleosome occupancy between stages. Furthermore, as chromatin compaction (and possibly other factors) could affect the efficiency of digestion towards mono-nucleosomes, we sought to retain all sizes of nucleosomal fragments in our final sequencing libraries. Therefore, we omitted the gel-size selection step from the sequencing library preparation procedure similar to what has been done previously by Henikoff and co-workers (10). Despite all these precautions, quantification of nucleosome occupancy in genomic regions with different digestion rates is difficult and statements on subtle differences in nucleosome occupancy between these regions are unlikely to be reliable. Consequently, we refrain from making definitive statements on possible subtle differences in nucleosome occupancy between *P. falciparum* coding and intergenic regions, but can say that large variation in nucleosome occupancy between these regions as reported by others (under much less controlled conditions (11-13)) is not observed in our dataset (see below for details).

Reproducibility

In order to differentiate technical noise from true biological differences between MNase-Seq samples, we included a technical replicate control. T40 stage nuclear pellets from the same biological collection were independently digested and further processed for sequencing to generate libraries T40A and T40B (Supplementary Figure 1). These libraries were used to assay inter-sample variation resulting from technical causes/noise. Since these libraries displayed high reproducibility of MNase-seq signal, they were pooled *in silico* and used as combined T40 sample for most analysis.

gDNA control

In order to as much as possible correct for technical artefacts introduced during library preparation, amplification and sequencing we introduced a genomic DNA control, although it does not correct for problems introduced during the digestion phase of the process. As an alternative, MNase-digested gDNA could have been employed. However, it is commonly argued that such a control would not correct for MNase-mediated digestion bias appropriately. Rather, digestion of naked DNA by MNase (or other nucleases) will likely exaggerate their sequence preference and will not be representative of the cutting preferences observed in a chromatin context where access to DNA is much more limited (14). Importantly, however sonicated gDNA control does correct for many other influences that would lead to incorrect conclusion and therefore is a vital addition to this dataset.

Nucleosome occupancy in intergenic regions and coding sequences are largely comparable

By far the most obvious discrepancy between our data and previous nucleosomes positioning datasets (11-13) relates to the occupancy of nucleosomes in intergenic regions. While earlier studies claim an on average 5-10 fold lower occupancy in intergenic regions compared to coding sequences, we have never detected such marked difference ((1,15), this study). Importantly, the nucleosome occupancy profiles in earlier studies, as well as in our uncorrected profiles (Supplementary Figure 3B) shows marked correlation to the GC-content of the underlying DNA. Since, as outlined above, virtually every step in the preparation of MNase-seq libraries can lead to artificial underrepresentation of AT-rich sequences it is therefore plausible that the lower MNase-seq signal in AT-rich intergenic regions is due to technical impediments. For example, the use of a gDNA control, which corrects for all biases associated with library preparation, sequencing and mapping of sequence reads, markedly reduces this bias (compare Figure 3A and Supplementary Figure 3B). (To this end it is pertinent to realize that the plot depicting our earlier native MNase-seq data in Figure 2C from (11) and is claimed to show ‘reduced (nucleosome) coverage on intergenic regions as compared to coding regions’ is not corrected for the corresponding gDNA control and instead of confirming reduced intergenic nucleosome occupancy, it only depicts the anti-AT-bias know to occur with version2/3 Illumina sequencing reagents.) But even if a gDNA control is used, several other experimental steps or a combination thereof can lead to depletion of the MNase-seq signal in intergenic sequences. For example, incubating or de-cross-linking at high temperature (12,13), (over)digestion of chromatin below mono-nucleosomal fragment size (11) or ChIP (11,13) can all contribute to such an effect.

Besides these technical arguments, we also have some theoretical problems with severely reduced occupancy of nucleosomes in intergenic regions. For example it is difficult to imagine how a 23Mb genome with only intermittent occupancy of nucleosomes maintains its integrity and is segregated appropriately during schizogony. Furthermore, such reduced level of occupancy (i.e. 5-10 fold) in regulatory sequences is in our view incompatible with strong positioning of nucleosomes in these regions and their function in gene expression regulation. Consequently, we argue that occupancy of nucleosomes is not markedly different between intergenic and coding sequences of the *Plasmodium falciparum* genome, similarly what has been reported for another AT-rich genome (16).

Relation between nucleosome occupancy and gene expression

A relation between nucleosome occupancy and expression level has been claimed earlier by Bunnik and colleagues (11). By analysing the average nucleosome occupancy in the promoter (defined as 500bp upstream of the ATG) as well as coding region of groups of genes with different mRNA abundance, they suggested that nucleosome occupancy decreases in promoter regions, while increases in coding bodies as a function of the transcriptional activity. (This latter finding contrasts reports from model organisms were highly transcribed genes display a subtle reduction in their coding body nucleosome occupancy (9)). In our analysis we could not reliably detect such correlation (data not shown). However, earlier we observed that highly expressed genes tend to possess an AT-richer promoter (17), and GC-richer coding sequence (unpublished observation). Therefore, it is not unthinkable that the above observation is a mere consequence of the strong correlation between GC-content and nucleosome occupancy in this dataset (11). On the other hand, even after correcting for AT-related biases, we find that the size of local, nucleosome-depleted region right in front of the TSS clearly correlates with the mRNA abundance (Figure 3). Furthermore, we show that dynamic eviction of nucleosomes occurs at or right next to the TSS at stages when the mRNA abundance of the corresponding gene is the highest, suggesting that the local depletion we observe is either a cause or consequence for temporal activation of the gene

Global nucleosome occupancy changes during development are difficult to assess

Profiling of the nucleosome landscape at 8 stages enabled detailed analysis of local chromatin dynamics during intraerythrocytic development. Notably, analysis of bulk nucleosome occupancy changes between the stages would have required additional sophisticated controls (i.e. co-digestion of a fixed amount of spiked-in chromatin from another species alongside the chromatin of *P. falciparum*), which we did not include. Therefore, we refrain from making conclusions on overall nucleosome occupancy differences during asexual cycle progression. We also cannot support the conclusion of Bunnik and colleagues (11) on substantially reduced overall nucleosome occupancy at the trophozoite stage, as this claim has been made in absence of such control. Instead, we concentrate our analysis on local rather than global changes (see Discussion main text) and want to emphasize that caution needs to be applied with respect to statements on (global) nucleosomes occupancy differences.

Supplementary Materials & Methods:

Parasite Culture

3D7 *Plasmodium falciparum* blood-stage parasites obtained from the Swiss Tropical and Public Health Institute (Basel, Switzerland - a kind gift from Dr. T. Voss) were cultured in standard RPMI medium supplemented with 10% human serum and 0.2% NaHCO3 in 2.5% human O^+ red blood cells. 75 cm^2 tissue culture flasks were incubated at 37ºC in candle jars under low oxygen conditions. Multiple rounds of panning were performed as described in (18) with the following modifications, to obtain a parasite population homogenously expressing a single *var* gene (*var2csa*, PF3D7_1200600). In short, CSA coated plates were blocked using 1% casein sodium salt from bovine milk (Sigma C8654-500G) solution and washed three times with RPMI media before parasites were allowed to bind for 30 min at 37 ºC without agitation followed by 5 RPMI washes. Bound parasites were collected in RPMI media supplemented with serum and NaHCO3 and allowed to grow to ~5-10% parasitemia before the next round of panning was performed. Preferential expression of *var2csa* and silencing of all other *var* genes was verified by RT-qPCR prior to synchronization with multiple rounds of sorbitol treatments. After a final Percoll gradient centrifugation human red blood cells were added with a few hours delay as in (1), to prevent "premature" invasion and therefore ensure better synchronicity of the culture. The blood was pre-filtered twice over sterile PlasmoPure (EuroProxima) filters to remove human white blood cells. T0 was set as the time when the first invasions were observed, resulting in an ~8 hour synchronicity window for >95% of parasites. Cross-linked nuclei (for MNase-Seq) and RNA (for RNA-seq) were collected every 5 hours. Medium was changed at least every 10 hours, but not within 10 hours from collection. For T25 and T30 collections culture volume was doubled at the T9, for T35 and T40 collections at the T18 media change, respectively. Cultures were divided over separate culture flasks (20ml each), but mixed upon every media change. Importantly, cross-linked nuclei and RNA were collected from the pooled culture flasks of the same batch of synchronized parasites to ensure completely matched nucleosome-positioning and transcriptome profiles. Parasite staging is comparable to Figure S1B of (1).

MNase-Seq and pellet control

Formaldehyde was immediately added to 37 ºC parasite cultures to a 1% final concentration, followed by 15 min incubation at 37 ºC while shaking. Cross-linking reaction was quenched by addition of 0.125M glycine (final concentration) and all subsequent steps were performed at 4 ºC unless noted otherwise. Parasites were isolated from red blood cells by 0.05% saponin lysis, followed by the isolation of parasite nuclei on a 0.25 M sucrose cushion using cell lysis buffer (10 mM Tris pH8, 3 mM $MgCl_2$, 0.2% NP40, Roche Protease Inhibitor Cocktail). Isolated nuclei were divided over multiple aliquots, which were used to determine the optimal digestion time for each time-point (as judged by comparable digestion of different time-points on agarose gel (Figure 1B)). Enzymatic digestion of the chromatin was performed in digestion buffer (50 mM Tris pH7.4, 4 mM $MgCl_2$, 1mM $CaCl_2$, 0.075% NP40, 1mM DTT, Roche Protease Inhibitor

Cocktail) with 0.5 U MNase (Worthington Biochemicals Corporation) and 100 U Exonuclease III (New England Biolabs) in 150 µl aliquots for 3.5-14 min at a 37 ºC waterbath with regular agitation. Digestion reactions were stopped by addition of 1 reaction volume quenching solution (2% Triton X100, 0.6% SDS, 300 mM NaCl, 6 mM EDTA, Roche Protease Inhibitor Cocktail) and placed at 4 ºC. Nuclei were mildly sonicated for 6 x 10 sec (setting low, Bioruptor™ Next Gen, Diagenode) to free the cross-linked nucleosomes from the nuclear membranes and the supernatant containing soluble chromatin was collected after centrifugation for 10 min at 9800 x g (this is the X-linked chromatin used for MNase-Seq and α-histone H4 ChIP reactions). Nuclear pellets were decrosslinked (see conditions below) to assess whether the material residing with the nuclear membranes is equally distributed over the genome or enriched for certain genomic regions (pellet control). DNA yield from the pellet of each time-point was quantified and DNA size distribution was confirmed to be marginally higher in size-range to MNase digested soluble chromatin (Supplementary Figure 1). Since for the T15 time-point the highest percentage of chromatin remained with the nuclear membranes, this sample was selected for sequencing as the pellet control. MNase-Seq chromatin and nuclear pellets were decrosslinked overnight at a 45 ºC shaking heatblock in decrosslinking buffer (1% SDS, 0.1 M $NaHCO_3$, 1 M NaCl) after which DNA was isolated via QIAquick column purification (Qiagen). Digestion efficiency was verified on 2% agarose gel (Figure 1B). Importantly, to enable, as much as possible, differentiation of technical variation from biological variation between the different samples, a technical replicate was included for the T40 time-point, where two different aliquots from the same nuclear collection were independently digested and processed for MNase-Seq. These technical replicates were called T40A and T40B and for most analysis sequence reads from both samples were combined (T40 sample).

Chromatin Immunoprecipitation

ChIPs were performed using either 200ng X-linked T40-stage chromatin (from sample T40A) for ChIP sequencing or 80ng X-linked chromatin from each stage for ChIP-qPCR. Chromatin was incubated with 1 µg α-H4 core antibody (Abcam Ab17036, lot GR8733-1) or 1 µg normal rabbit IgG control antibody (ChIP sequencing only, Upstate 12-370, lot DAM1421465) in ChIP buffer (20 mM Tris pH8, 2 mM EDTA, 1% Triton-X100, 0.15% SDS, 150 mM NaCl, Roche Protease Inhibitor Cocktail) O/N while rotating at 4 °C. 10 µl ProtA Dynabeads (Life Technologies 10008D) and 10 µl ProtG Dynabeads (Life Technologies 10009D) were added to each reaction and allowed to rotate for another 2 hours at 4 °C, after which ChIPs were washed with 400 µl wash buffer: 1x ChIP wash 1 (20 mM Tris pH8, 2 mM EDTA, 1% Triton-X100, 0.1% SDS, 150 mM NaCl), 2x ChIP wash 2 (20 mM Tris pH8, 2 mM EDTA, 1% Triton-X100, 0.1% SDS, 500 mM NaCl), 2x ChIP wash 3 (10 mM Tris pH8, 1 mM EDTA). Reactions were rotated for 5 min at 4 °C in between each wash. Immunoprecipitated chromatin was eluted in elution buffer (1% SDS, 0.1M $NaHCO_3$) during 20 min rotation at RT and decrosslinked as described above (“MNase-Seq and pellet control” section). qPCRs were performed on MNase-digested chromatin before and after α-histone H4 ChIP, respectively. For each primer pairs relative

value was calculated in relation to a genomic DNA dilution series. Relative abundance (MNase-digested chromatin) or relative recovery (α-histone H4 ChIP) was calculated by dividing the value for the dynamic region by the value for the respective control region.

Genomic DNA extraction and fragmentation

Genomic DNA was extracted from sorbitol synchronized ring stage 3D7 *Plasmodium falciparum* cultures of the same strain as was used for MNase-Seq. Cultures were PlasmoPure (EuroProxima) filtered before collection. Native nuclei collection, proteinase K treatment and genomic DNA isolation were performed as described in (1) with the following modifications. An RNaseA incubation step ensured degradation of RNA. gDNA was fragmented using a Bioruptor^{TM} Next Gen (Diagenode) to a ~100-400 bp range.

Sequencing library preparation

Since our linear amplification protocol (LADS, (19)) was difficult to adapt to the NextFlex adapters of Illumina sequencing we used an optimised KAPA protocol for library preparation with comparable performance (as long as proper controls are included to correct for the remaining PCR bias). 10 ng of MNase-Seq inputs, gDNA control, T15 pellet control and 5.7 ng of T40 α-histone H4 ChIP DNA were used to generate sequencing libraries. All samples were first end repaired, a 3' A-overhang added and NextFlex barcoded adapters (Bio Scientific) were ligated as described in. Adapter ligation was followed by 2 successive Agencourt AMPure XP bead purifications (Beckman Coulter) and library amplification by an in-house *Plasmodium*-optimized KAPA PCR amplication protocol. 2x KAPA HiFi HotStart ready-mix (KAPA Biosystems) and NextFlex primers were used for 9 cycles of PCR amplification using the following conditions: 98 ºC for 2 min, (9 cycles of: 98 ºC for 20 sec, 62 ºC for 3 min), 62 ºC for 5 min. Libraries were again Agencourt AMPure XP bead purified before sequencing. Importantly, no size-selection step was applied to the MNase-Seq and control libraries allowing assessment of the full range of DNA fragments resulting from enzymatic chromatin digestion and *in silico* size-selection of paired-end sequenced libraries.

RNA isolation, cDNA synthesis and preparation of strand-specific RNA-seq libraries

Total RNA isolation and oligo-dT-selection to enrich for polyA^{+} mRNA were performed as described elsewhere (20) with addition of an extra in-solution DNase treatment (TURBO DNase, Ambion) to remove contaminating gDNA. Integrity of total RNA was confirmed on 1.5% agarose gel and gDNA contamination was tested by qPCR. 2 µg of total RNA-equivalent polyA^{+} mRNA was fragmented by hydrolysis as described in (20). cDNA synthesis was modified as follows from (20) to allow maintenance of directional information for strand-specific RNA-seq. During first strand synthesis 0.2 µg Actinomycin D was included in the reaction and a 15 min 70 ºC enzyme deactivation step was included after first strand synthesis followed by QIAquick MinElute purification (Qiagen) of the

first strand cDNA. During second strand synthesis dTTP was replaced by dUTPs, resulting in incorporation of U-bases instead of T-bases in the second cDNA strand only. 4 ng double-stranded cDNA was used for sequencing library preparation as described above ("Sequencing library preparation" section) with the following adaptation: after adapter ligation a 15 min incubation at 37 ºC with USER enzyme (New England Biolabs) ensured degradation of the second cDNA strand specifically (by degrading the U-base containing strand) thereby retaining directional information for sequencing. Subsequently, 4 cycles of *Plasmodium*-optimized kapa pre-PCR (for details see "Sequencing library preparation" section) were performed, followed by size-selection of 200-300bp cDNA fragments and subsequent amplification of another 9 cycles of *Plasmodium*-optimized kapa PCR resulting in total 13 cycles of PCR amplification.

High throughput sequencing

MNase-Seq, gDNA, T40A α-histone H4 ChIP and T15 pellet control samples were sequenced on a HiSeq 2000 system (Illumina) to obtain 100bp paired-end reads (TruSeq PE Cluster Kit v3, Sequencing reagents v3). Directional RNA-seq libraries were sequenced for 92 cycles single-end (TruSeq SR Cluster Kit v3, Sequencing reagents v3) on the same system.

Native nuclei isolation, nuclear extract generation and DNA pulldowns

3D7 *Plasmodium falciparum* parasites were cultured as described above and mixed-stage native nuclei were collected on a 0.25M sucrose cushion as in (15), with the following modifications: Infected-RBC cultures were filtered over PlasmoPure (EuroProxima) filters to remove human white blood cells and all (wash) buffers including and after saponin lysis of RBCs contained Protease Inhibitor Cocktail (Roche). To increase efficiency of parasite retrieval, supernatants were spun twice in the wash steps following saponin lysis. Isolated nuclei were resuspended in Cell Lysis Buffer (10mM Tris pH8.0, 3mM $MgCl_2$, 0.2% NP40, Protease Inhibitor Cocktail) supplemented with 20% glycerol and pelleted by centrifugation (15min, 3500 x g, 4 ºC) after which nuclear pellets were snap-frozen and stored at -80 ºC. Upon thawing, isolated nuclei were washed once with buffer (50mM Tris pH7.4, 4mM $MgCl_2$, 1mM $CaCl_2$, Protease Inhibitor Cocktail) and pelleted by centrifugation for 13 min at 1500 x g. Supernatant was removed and pellet volume determined as accurately as possible. Nuclei were resuspended in 6 pellet volumes of High Salt Extraction Buffer (50mM HEPES pH7.5, 20% glycerol, 420mM NaCl, 1.5mM $MgCl_2$, 1mM DTT, 0.4% NP40, Protease Inhibitor Cocktail – modified from (21)), nuclear membranes ruptured by 15-20 strokes of an eppendorf-douncer and proteins extracted by rotation for 2 hours at 4 ºC. Subsequently, samples were spun 10min at 17000 x g and nuclear extract was collected. To enhance efficiency of protein extraction a second extraction was performed on the pelleted nuclei using 4 original-pellet volumes of High Salt Extraction Buffer, 2 hours rotation at 4 ºC followed by spinning for 10min at 17000 x g. The second extract was collected and pooled with the first extract, after which nuclear extracts were quantified, snap-frozen and stored at -80 ºC. Two independent batches of

mixed-stage asexual extracts were used for pulldowns consisting of ~66% Rings, ~27% Trophozoites, ~7% Schizonts (Experiment 1, probe PF3D7_1312700 and probe PF3D7_0114000) and ~62% Rings, ~29% Trophozoites, ~9% Schizonts (Experiment 2, probe PF3D7_1312700 only) respectively.

DNA pulldown procedure was modified from (22). Probes were ordered as single stranded purified DNA with the forward oligo containing a 5' Biotin-TEG modification and the reverse oligo being unmodified (biomers.net, probe sequences are indicated in Supplementary Table 3). Oligo's were annealed using 1.5 molar ratio of reverse over forward oligo in annealing buffer (10mM HEPES pH8.0, 500mM NaCl, 1mM ETDA) in a PCR machine set at initial heating at 95 ºC for 2min followed by a temperature decrease of 0.1 ºC/s until 4 ºC, with pause-steps of 2min when temperatures of 75 ºC, 65 ºC, 50 ºC, 37 and 20 ºC were reached. Subsequently, oligo's were diluted in DNA Binding Buffer (DBB; 10mM HEPES pH8.0, 1M NaCl, 10mM EDTA, 0.05% NP40), aliquoted and stored at -20C. 5ul High-performance streptavidin-sepharose beads (GE Healthcare, 17511301) were washed once with DBB, resuspended in 350ul DBB containing 50pmol of double-stranded biotinylated-DNA oligo and DNA was allowed to bind for minimally 1h while rotating at RT. Subsequent steps were performed at 4 ºC. Beads were washed twice with 500µl DBB and twice with 500µl Protein Binding Buffer (PBB; 50mM HEPES pH8.0, 150mM NaCl, 0.1% NP40, 1mM DTT, Protease Inhibitor Cocktail (Roche)). Prior to addition of 230-500µg of nuclear extract to the beads, nuclear extracts were diluted and competitor was added to obtain the following composition (50mM HEPES pH7.5, 10% glycerol, 150mM NaCl, 1.5mM $MgCl_2$, 1mM DTT, 0.125% NP40, Protease Inhibitor Cocktail (Roche), 6ng/µl single-stranded yeast RNA, 6ng/µl poly (dI:dC) and 6ng/µl poly (dA:dT) competitor) and diluted nuclear extract was centrifuged for 25min at 17000 x g to remove precipitations. Then 500-550µl of diluted NE was incubated with the beads while rotating for 1.5h. To remove unbound proteins, beads were washed once with 1ml PBB, twice with 1ml PBB without protease inhibitor and twice with wash buffer without NP40 (50mM HEPES pH8.0, 150mM NaCl) to remove detergents that can interfere with mass spectrometric analysis. Protein reduction, alkylation and digestion with Trypsin/LysC was performed in eppendorf tubes on-beads as in (22). Subsequently peptides were chemically labelled using the dimethyl-labelling approach (23), samples were pooled and purification on stage-tips as described previously (24). DNA pulldowns were performed in duplicate on the same nuclear extract using label-swap conditions.

Mass Spectrometry and MS data analysis

Samples were eluted from stage-tips and analysed on a QExactive mass spectrometer (Thermo Fisher Scientific) as in (22). In short, 1/3 of the sample pool was separated over a 30cm C18-reverse phase column (1.8µm Reprosil-Pur C18-AQ, dr. Maisch 9852) and eluted over a 94min gradient (5.6% acetonitrile/0.1% formic acid – 25.6% acetonitrile/0.1% formic acid) using an Easy-nLC 1000 (Thermo Fisher Scientific) and directly injected into the mass spectrometer. Data was acquired in TOP10 data-dependent acquisition mode with dynamic exclusion enabled for 20s. Resolution for MS was set at 70.000 at m/z = 400 and for MS/MS at 17.5000.

Raw MS spectra were analysed by MaxQuant (version 1.4.1.2) using standard settings (25) with the following modifications. Multiplicity was set at 2, adding a mass of 28.03Da (light-labelled) or 32.06 Da (heavy-labelled) to the peptides N-terminus and lysine residues. Methylthiol (added mass of 45.99Da) was specified as fixed modification of cysteine amino acids and acetylation of N-termini and oxidation of methionines were included as variable modifications. Specific enzyme cleavage by Trypsin/P was selected, with a maximum of two missed cleavages allowed. The match-between-runs option was activated (using standard settings) and calculation of iBAQ values was enabled. Peptide masses were searched against the *Plasmodium falciparum* 3D7 annotated proteome (PlasmoDB9.3) with the entire human proteome included in the contaminants list using the integrated Andromeda search engine. Mass tolerance was set at 4.5 ppm for precursor ions and 20 ppm for fragment ions, and peptides and proteins were accepted with a 0.01 FDR cut-off. Protein quantification required minimally a single peptide-ratio.

Downstream analysis was performed using the Perseus software package (version 1.4.0.20) (26). Normalized H/L-ratio's were log2-transformed and used for downstream analysis. A log10-transformation was applied to intensity values, the label-swap situation was reverted and the protein list was filtered for contaminants and reverse hits. Significant outliers were determined using the intensity-based Significance B option (double-sided Benjamini-Hochberg statistical test) with a FDR cut-off set at 0.05 for both forward and reverse experiments and proteins required a minimum of 2 peptides of which at least 1 unique in order to be identified as significant hit. Data was plotted in R and significant outliers were labelled.

High-throughput sequencing data analysis

Data processing and analysis was done with BioPerl (Version 1.6.9) (27), BioRuby (Version 1.4.3.0001) (28), rtracklayer (Version 1.20.4) (29), samtools (Version 0.1.19) (30), bedtools (Version 2.20.1) (31), Picard (Version 1.101) (32).

Single-End RNA-seq Read Mapping and Transcript Quantification

The single-end RNA-seq samples were mapped with BWA (Version 0.6.2-r126, default parameters) to either the complete *P. falciparum* assembly for the genomic tracks or the spliced transcriptome for transcript quantification (PlasmoDB 6.1) and filtered to mapping quality ≥15. Between 13.9 and 24.9 million reads mapped to transcripts (Supplementary Table 1).

The total RNA level in *P. falciparum* undergoes substantial changes during the intraerythrocytic development and therefore normalisation of the expression data based on total RNA abundance and nuclei number might better approximate the real transcriptional dynamics of individual genes (1). We therefore analysed our RNA-seq data both with standard RPKM expression estimates and rescaled RPKM expression values (see Supplementary Table 4 for the scaling constants).

Paired-End MNase-Seq Read Mapping

Paired-end reads were clipped to 72bp and mapped with BWA (Version 0.6.2-r126) to the PlasmoDB 6.1 assembly. We decided to trim MNase-seq reads to 72bp, to be able to map sub-nucleosomal fragments shorter than 100bp, but retain decent mappability of the sequence reads. The paired-end mapping was done such that up to 1000 alternative alignment positions were reported for each read (bwa sampe –n 1000). Subsequently, read pairs aligning with mapping quality below 30 or aligning to more than a single position with alignment pair distances below 1 kb were discarded using a custom script. Between 26.5 and 41.4 million paired-end fragments (mf) were mapped per time-point (Supplementary Table 1).

Genomic DNA Control

As a means to control for amplification and sequencing biases we sequenced a sonicated genomic DNA sample, which unavoidably has a different distribution of fragment lengths than MNase-digested and nucleosome-protected DNA. To avoid potential biases associated with such differences in the insert-size distributions (ISD), we transformed the ISD of the gDNA control using a sampling approach. For every MNase-seq sample we created a resampled gDNA control with a matching ISD. To that aim, for every insert-size the number of fragments was calculated based on the target ISD and a desired total number of 150 million fragments. Fragments were then randomly selected from the fragments of that length out of the 274 million gDNA fragments. The decision to sample 150 million fragments was based on the observation that with this number of fragments for most MNase-seq samples none of the relevant insert sizes (75-339 bp) were over-sampled by a factor larger than 3.

Tracks and Profiles

For the genome profiles the normalized coverage per site (n_i) was calculated as $n_i = r_i \times g/b$, where r_i is the number of fragments covering the sites i in the sample, b is the total number of bases in fragments, and g is the genome size. Thus, a normalized coverage value of 1 represents the coverage expected if fragments are randomly distributed across the genome. For single-end RNA-seq data we used the read length (92bp) as fragment length to generate tracks. To obtain MNase-seq coverages corrected for amplification and sequencing biases the pair-wise ratios of the normalized MNase-seq and the matching normalized re-sampled gDNA control tracks were calculated. To avoid division by zero an offset of 1e-3 was added to both enumerator and denominator. For the average profile plots of ratios the normalized signals of all sites were first averaged and then divided.

A/T homo- and heteropolymeric runs

Runs of at least 6 bases of AT- or TA-dinucleotide repeats and A- or T-homopolymeric stretches were identified in the *P. falciparum* assembly using the regular expressions /^(AT){3,}A?|(TA){3,}T$/ and /^(A{6,}|T{6,})$/, respectively. The run-frequency profiles represent the numbers of sites contained in such runs at given distances around fragment midpoints.

Upstream and Downstream Regions

The analysis of the sizes of intergenic regions suggests that upstream regions are about three times the size of downstream regions (17). We therefore assigned half of each convergent and divergent intergenic region to the neighbouring genes as downstream or upstream regions, respectively. Intergenic regions between co-oriented genes were split such that ¾ were assigned as upstream region to the one gene while ¼ was assigned to the other as downstream region. Additionally, upstream/downstream regions larger than 3 kb were shortened to 3 kb.

Landmark Sites

Gene ends (ATG, STOP) and splice sites were extracted from the PlasmoDB 6.1 annotation. The publication by Siegel *et al.* (33) provided a list of polyadenylation sites (PAS) for the *P. falciparum* 3D7 assembly v3, January 2012 (Sanger). First we selected a single TSS for each genes with the highest tag count. These were mapped to the PlasmoDB 6.1 assembly using the gene ID mapping table PlasmoDB-11.0_Pfalciparum3D7_GeneAliases.txt from the PlasmoDB website and the distance to the major PAS as offset to the gene end in the PlasmoDB 6.1 assembly.

Transcription start site identification

Total RNA was isolated from asynchronous *in vitro* cultured *P. falciparum* 3D7 parasites (using standard methods), and polyA+ RNA was isolated using magnetic oligo-d(T) beads. Superscript II reverse transcriptase was used to synthesise first strand cDNA using oligo-d(T) primers and in the presence of template switching oligos, which had the same sequence as those in the Smart-seq2 protocol (34). The resulting cDNA was fragmented, made into RNA-seq libraries using an amplification-free protocol (as described in (35)) and sequenced using 150bp paired-end reads on an Illumina MiSeq. Reads were mapped with SMALT using soft-clipping option (www.sanger.ac.uk/resources/software/smalt/), and reads mapping as proper pairs were selected. Reads containing the sequence corresponding to the template-switching oligo were identified using BLAT. The most prominent TSSs were identified by selecting the position (1bp window) with the highest number of 5'-ends mapping to the template strand in a maximum of 2kb intergenic region upstream of each gene (a minimum tag count of 10 was applied). A similar approach was used to identify genes with a second prominent TSS ("2nd TSS"), further than 100bp away from the most prominent TSS (Note that most 5' enriched RNA-seq tags localize within 100bp from the most prominent TSS, Supplementary Figure 5). We identified 620 secondary TSSs after filtering for at least 10 reads and 25% of the coverage of the most prominent TSS supporting at these sites. The predicted primarily and secondary TSSs are listed in Supplementary Table 5.

Heatmaps

Heatmaps of log2 MNase-Seq/gDNA ratio's were aligned on the position of landmark sites (TSS, ATG, splice donor, splice acceptor, STOP, TTS) and plotted using the R-packages ggplot2 (V.1.0.0), gtable (V.0.1.2) and ggdendro (V.0.1-15). Regions were ordered by mRNA abundance of the corresponding gene or distance to the neighbouring genomic feature, respectively. Profiles in Supplementary Figure 4A were topologically sorted guided by an average linkage clustering using the inverse Spearman correlation as distance measure (d=1-rS). Ten-thousand non-overlapping random sites were included as control in each plot.

Heatmaps of ApiAP2 mRNA steady-state abundance (Figure 5G) depict log2(Cy5/Cy3) ratio's throughout the 48h asexual intra-erythrocytic cycle measured by Bozdech and co-workers (36) and are visualized using plotly (https://plot.ly/).

Dynamic Sites

Three different filters were applied to identify dynamically changing 75bp (1/2 nucleosome) windows based on fragment midpoints counts per window. First, windows with coverage of ≤10 fragment midpoints at all 8 time-points were discarded. Second, windows with a variability of the normalized window coverage lower than 20% of the average coverage were discarded. Third, windows with noisy or rugged coverage profiles were discarded by restricting to the top 50% auto-correlating windows. For this we assumed a closed intra-erythrocytic cycle and calculated the auto-correlation as Pearson correlation between the vectors $(n_5, \dots, n_{40})$ and $(n_{40}, n_5, \dots, n_{35})$, where n_i denotes the normalized number of fragment midpoints at time-point i. Normalization of the midpoint counts was done by dividing by the total number of aligned fragments in the sample. Dynamic windows were assigned to a particular genomic region (e.g. 5' or 3' intergenic region) if more than half of the window overlap the region.

DNA motif analysis

We obtained positional probability matrices (PPMs) describing the putative TFBSs of PF10_0075 (PF3D7_1007700; motif for AP2-domain D3 only), PF13_0235 (PF3D7_1342900) (37) and PF14_0633 (PF3D7_1466400) (38). The PPMs were restricted to the central, highly informative core motifs (PF10_0075_D3|8-15; PF13_0235|8-14; PF14_0633|4-11) and used to detect the binding sites with MEME-FIMO Version 4.8.1.(39). Plots were smoothened using the R-function loess (family=gaussian, span=0.09). Positional probability matrices of core DNA-binding-motifs for AP2 proteins PF3D7_1107800 (from (37)) and PF3D7_1466400 (from (38)) were visualized using enoLOGOS (40).

Nucleosome Calling

DANPOS (Version 2.2.1; dpos, paired=1, nor=F) (41) was used to call nucleosomes with BAM files of all MNase-Seq samples (T5-T40) as foreground, each with an insert-size-

matched gDNA control BAM file as background. We selected “positioned nucleosomes” by restricting to sites called with a DANPOS fuzziness score < 35 and from these selecting the 10000 sites with the highest DANPOS summit value. (Positioned) nucleosomes were assigned to a genomic region if more than half of the underlying sequence fall into the region.

Supplementary References:

1. Bartfai, R., Hoeijmakers, W.A., Salcedo-Amaya, A.M., Smits, A.H., Janssen-Megens, E., Kaan, A., Treeck, M., Gilberger, T.W., Francoijs, K.J. and Stunnenberg, H.G. (2010) H2A.Z demarcates intergenic regions of the plasmodium falciparum epigenome that are dynamically marked by H3K9ac and H3K4me3. *PLoS Pathog*, **6**, e1001223.
2. Wal, M. and Pugh, B.F. (2012) Genome-wide mapping of nucleosome positions in yeast using high-resolution MNase ChIP-Seq. *Methods Enzymol*, **513**, 233-250.
3. Hoeijmakers, W.A., Flueck, C., Francoijs, K.J., Smits, A.H., Wetzel, J., Volz, J.C., Cowman, A.F., Voss, T., Stunnenberg, H.G. and Bartfai, R. (2012) Plasmodium falciparum centromeres display a unique epigenetic makeup and cluster prior to and during schizogony. *Cell Microbiol*, **14**, 1391-1401.
4. Das, P.M., Ramachandran, K., vanWert, J. and Singal, R. (2004) Chromatin immunoprecipitation assay. *Biotechniques*, **37**, 961-969.
5. Orlando, V., Strutt, H. and Paro, R. (1997) Analysis of chromatin structure by in vivo formaldehyde cross-linking. *Methods*, **11**, 205-214.
6. Chung, H.R., Dunkel, I., Heise, F., Linke, C., Krobitsch, S., Ehrenhofer-Murray, A.E., Sperling, S.R. and Vingron, M. (2010) The effect of micrococcal nuclease digestion on nucleosome positioning data. *PLoS One*, **5**, e15754.
7. Nikitina, T., Wang, D., Gomberg, M., Grigoryev, S.A. and Zhurkin, V.B. (2013) Combined micrococcal nuclease and exonuclease III digestion reveals precise positions of the nucleosome core/linker junctions: implications for high-resolution nucleosome mapping. *J Mol Biol*, **425**, 1946-1960.
8. Brogaard, K., Xi, L., Wang, J.P. and Widom, J. (2012) A map of nucleosome positions in yeast at base-pair resolution. *Nature*, **486**, 496-501.
9. Moyle-Heyrman, G., Zaichuk, T., Xi, L., Zhang, Q., Uhlenbeck, O.C., Holmgren, R., Widom, J. and Wang, J.P. (2013) Chemical map of Schizosaccharomyces pombe reveals species-specific features in nucleosome positioning. *Proc Natl Acad Sci U S A*, **110**, 20158-20163.
10. Henikoff, J.G., Belsky, J.A., Krassovsky, K., MacAlpine, D.M. and Henikoff, S. (2011) Epigenome characterization at single base-pair resolution. *Proc Natl Acad Sci U S A*, **108**, 18318-18323.
11. Bunnik, E.M., Polishko, A., Prudhomme, J., Ponts, N., Gill, S.S., Lonardi, S. and Le Roch, K.G. (2014) DNA-encoded nucleosome occupancy is associated with transcription levels in the human malaria parasite Plasmodium falciparum. *BMC Genomics*, **15**, 347.
12. Ponts, N., Harris, E.Y., Prudhomme, J., Wick, I., Eckhardt-Ludka, C., Hicks, G.R., Hardiman, G., Lonardi, S. and Le Roch, K.G. (2010) Nucleosome landscape and control of transcription in the human malaria parasite. *Genome Res*, **20**, 228-238.
13. Westenberger, S.J., Cui, L., Dharia, N. and Winzeler, E. (2009) Genome-wide nucleosome mapping of Plasmodium falciparum reveals histone-rich coding and histone-poor intergenic regions and chromatin remodeling of core and subtelomeric genes. *BMC Genomics*, **10**, 610.

14. Kaplan, N., Hughes, T.R., Lieb, J.D., Widom, J. and Segal, E. (2010) Contribution of histone sequence preferences to nucleosome organization: proposed definitions and methodology. *Genome biology*, **11**, 140.
15. Salcedo-Amaya, A.M., van Driel, M.A., Alako, B.T., Trelle, M.B., van den Elzen, A.M., Cohen, A.M., Janssen-Megens, E.M., van de Vegte-Bolmer, M., Selzer, R.R., Iniguez, A.L. *et al.* (2009) Dynamic histone H3 epigenome marking during the intraerythrocytic cycle of Plasmodium falciparum. *Proc Natl Acad Sci U S A*, **106**, 9655-9660.
16. Chang, G.S., Noegel, A.A., Mavrich, T.N., Muller, R., Tomsho, L., Ward, E., Felder, M., Jiang, C., Eichinger, L., Glockner, G. *et al.* (2012) Unusual combinatorial involvement of poly-A/T tracts in organizing genes and chromatin in Dictyostelium. *Genome Res*, **22**, 1098-1106.
17. Hoeijmakers, W.A., Salcedo-Amaya, A.M., Smits, A.H., Francoijs, K.J., Treeck, M., Gilberger, T.W., Stunnenberg, H.G. and Bartfai, R. (2013) H2A.Z/H2B.Z double-variant nucleosomes inhabit the AT-rich promoter regions of the Plasmodium falciparum genome. *Mol Microbiol*, **87**, 1061-1073.
18. Noviyanti, R., Brown, G.V., Wickham, M.E., Duffy, M.F., Cowman, A.F. and Reeder, J.C. (2001) Multiple var gene transcripts are expressed in Plasmodium falciparum infected erythrocytes selected for adhesion. *Mol Biochem Parasitol*, **114**, 227-237.
19. Hoeijmakers, W.A., Bartfai, R., Francoijs, K.J. and Stunnenberg, H.G. (2011) Linear amplification for deep sequencing. *Nat Protoc*, **6**, 1026-1036.
20. Hoeijmakers, W.A., Bartfai, R. and Stunnenberg, H.G. (2013) Transcriptome analysis using RNA-Seq. *Methods Mol Biol*, **923**, 221-239.
21. Bantscheff, M., Hopf, C., Savitski, M.M., Dittmann, A., Grandi, P., Michon, A.M., Schlegl, J., Abraham, Y., Becher, I., Bergamini, G. *et al.* (2011) Chemoproteomics profiling of HDAC inhibitors reveals selective targeting of HDAC complexes. *Nature biotechnology*, **29**, 255-265.
22. Hubner, N.C., Nguyen, L.N., Hornig, N.C. and Stunnenberg, H.G. (2015) A quantitative proteomics tool to identify DNA-protein interactions in primary cells or blood. *J Proteome Res*, **14**, 1315-1329.
23. Boersema, P.J., Raijmakers, R., Lemeer, S., Mohammed, S. and Heck, A.J. (2009) Multiplex peptide stable isotope dimethyl labeling for quantitative proteomics. *Nat Protoc*, **4**, 484-494.
24. Rappsilber, J., Mann, M. and Ishihama, Y. (2007) Protocol for micro-purification, enrichment, pre-fractionation and storage of peptides for proteomics using StageTips. *Nat Protoc*, **2**, 1896-1906.
25. Cox, J. and Mann, M. (2008) MaxQuant enables high peptide identification rates, individualized p.p.b.-range mass accuracies and proteome-wide protein quantification. *Nature biotechnology*, **26**, 1367-1372.
26. Cox, J. and Mann, M. (2012) 1D and 2D annotation enrichment: a statistical method integrating quantitative proteomics with complementary high-throughput data. *BMC bioinformatics*, **13 Suppl 16**, S12.
27. Stajich, J.E., Block, D., Boulez, K., Brenner, S.E., Chervitz, S.A., Dagdigian, C., Fuellen, G., Gilbert, J.G., Korf, I., Lapp, H. *et al.* (2002) The Bioperl toolkit: Perl modules for the life sciences. *Genome Res*, **12**, 1611-1618.

28. Goto, N., Prins, P., Nakao, M., Bonnal, R., Aerts, J. and Katayama, T. (2010) BioRuby: bioinformatics software for the Ruby programming language. *Bioinformatics*, **26**, 2617-2619.
29. Lawrence, M., Gentleman, R. and Carey, V. (2009) rtracklayer: an R package for interfacing with genome browsers. *Bioinformatics*, **25**, 1841-1842.
30. Li, H., Handsaker, B., Wysoker, A., Fennell, T., Ruan, J., Homer, N., Marth, G., Abecasis, G., Durbin, R. and Genome Project Data Processing, S. (2009) The Sequence Alignment/Map format and SAMtools. *Bioinformatics*, **25**, 2078-2079.
31. Quinlan, A.R. and Hall, I.M. (2010) BEDTools: a flexible suite of utilities for comparing genomic features. *Bioinformatics*, **26**, 841-842.
32. Alberts, I.L., Wang, Y. and Schlick, T. (2007) DNA polymerase beta catalysis: are different mechanisms possible? *J Am Chem Soc*, **129**, 11100-11110.
33. Siegel, T.N., Hon, C.C., Zhang, Q., Lopez-Rubio, J.J., Scheidig-Benatar, C., Martins, R.M., Sismeiro, O., Coppee, J.Y. and Scherf, A. (2014) Strand-specific RNA-Seq reveals widespread and developmentally regulated transcription of natural antisense transcripts in Plasmodium falciparum. *BMC Genomics*, **15**, 150.
34. Picelli, S., Bjorklund, A.K., Faridani, O.R., Sagasser, S., Winberg, G. and Sandberg, R. (2013) Smart-seq2 for sensitive full-length transcriptome profiling in single cells. *Nature methods*, **10**, 1096-1098.
35. Spence, P.J., Jarra, W., Levy, P., Reid, A.J., Chappell, L., Brugat, T., Sanders, M., Berriman, M. and Langhorne, J. (2013) Vector transmission regulates immune control of Plasmodium virulence. *Nature*, **498**, 228-231.
36. Bozdech, Z., Llinas, M., Pulliam, B.L., Wong, E.D., Zhu, J. and DeRisi, J.L. (2003) The transcriptome of the intraerythrocytic developmental cycle of Plasmodium falciparum. *PLoS biology*, **1**, E5.
37. Campbell, T.L., De Silva, E.K., Olszewski, K.L., Elemento, O. and Llinas, M. (2010) Identification and genome-wide prediction of DNA binding specificities for the ApiAP2 family of regulators from the malaria parasite. *PLoS Pathog*, **6**, e1001165.
38. De Silva, E.K., Gehrke, A.R., Olszewski, K., Leon, I., Chahal, J.S., Bulyk, M.L. and Llinas, M. (2008) Specific DNA-binding by apicomplexan AP2 transcription factors. *Proc Natl Acad Sci U S A*, **105**, 8393-8398.
39. Grant, C.E., Bailey, T.L. and Noble, W.S. (2011) FIMO: scanning for occurrences of a given motif. *Bioinformatics*, **27**, 1017-1018.
40. Workman, C.T., Yin, Y., Corcoran, D.L., Ideker, T., Stormo, G.D. and Benos, P.V. (2005) enoLOGOS: a versatile web tool for energy normalized sequence logos. *Nucleic Acids Res*, **33**, W389-392.
41. Chen, K., Xi, Y., Pan, X., Li, Z., Kaestner, K., Tyler, J., Dent, S., He, X. and Li, W. (2013) DANPOS: dynamic analysis of nucleosome position and occupancy by sequencing. *Genome Res*, **23**, 341-351.

Supplementary Table 1: Mapping statistics

Sample:	Total Reads [e]	SE reads mapped against genome[a,c]	SE reads mapped against transcriptome [a,d]	PE fragments mapped [b,c]	GEO accession number:
MNase-Seq T5	8.93E+07	NaN	NaN	3.83E+07	GSE66185: GSM1616484
MNase-Seq T10	7.70E+07	NaN	NaN	3.27E+07	GSE66185: GSM1616485
MNase-Seq T15	7.89E+07	NaN	NaN	3.32E+07	GSE66185: GSM1616486
MNase-Seq T20	9.78E+07	NaN	NaN	4.14E+07	GSE66185: GSM1616487
MNase-Seq T25	7.46E+07	NaN	NaN	3.11E+07	GSE66185: GSM1616488
MNase-Seq T30	9.42E+07	NaN	NaN	3.98E+07	GSE66185: GSM1616489
MNase-Seq T35	6.82E+07	NaN	NaN	2.91E+07	GSE66185: GSM1616490
MNase-Seq T40A	2.35E+07	NaN	NaN	1.00E+07	GSE66185: GSM1616491
MNase-Seq T40B	3.84E+07	NaN	NaN	1.65E+07	GSE66185: GSM1616492
gDNA control	6.74E+08	NaN	NaN	2.74E+08	GSE66185: GSM1616495
H4-ChIP control T40A	1.10E+08	NaN	NaN	4.74E+07	GSE66185: GSM1616493
Pellet-Seq control T15	2.70E+07	NaN	NaN	5.03E+06	GSE66185: GSM1616494
directional RNA-Seq T5	2.62E+07	1.84E+07	1.64E+07	NaN	GSE66185: GSM1616496
directional RNA-Seq T10	2.55E+07	1.79E+07	1.63E+07	NaN	GSE66185: GSM1616497
directional RNA-Seq T15	2.18E+07	1.61E+07	1.41E+07	NaN	GSE66185: GSM1616498
directional RNA-Seq T20	3.18E+07	2.36E+07	2.21E+07	NaN	GSE66185: GSM1616499
directional RNA-Seq T25	2.11E+07	1.54E+07	1.39E+07	NaN	GSE66185: GSM1616500
directional RNA-Seq T30	1.99E+07	1.50E+07	1.39E+07	NaN	GSE66185: GSM1616501
directional RNA-Seq T35	3.08E+07	2.19E+07	2.01E+07	NaN	GSE66185: GSM1616502
directional RNA-Seq T40	4.22E+07	2.75E+07	2.49E+07	NaN	GSE66185: GSM1616503

[a] MAPQ >= 15 filtered for SE reads
[b] MAPQ >= 30 filtered for PE reads
[c] mapped against P. falciparum 3D7 PlasmoDB6.1 reference genome
[d] mapped against P. falciparum 3D7 PlasmoDB6.1 reference transcriptome
[e] Of note, for PE sequenced libraries, the number of reads is twice as high as the actual number of fragments sequenced, as each fragment is sequenced from both sides.

Supplementary Table 3: Probes for DNA pull-down experiments

Probe name:	Sequence [a, b]:	Genomic location:
PF3D7_1312700	TTATAGGAATTATAATGTCAAACCC**TGCATGCA**TAATATAAGGAAAAATTCATATTCT [a]	Chr13:535585-535632
PF3D7_1312700_mut	TTATAGGAATTATAATGTCAAACCCgaccagttTAATATAAGGAAAAATTCATATTCT [b]	-
PF3D7_0114000	TATATATATAATATTTATTTATTTA**TGCATGCA**TGAATAATTTTTACTTTGAAAAATA [a]	Chr1:543777-543834
PF3D7_0114000_mut	TATATATATAATATTTATTTATTTAatctgagcTGAATAATTTTTACTTTGAAAAATA [b]	-

[a] Predicted PF3D7_1466400 motif is indicated in red bolded CAPITALS

[b] Mutated motifs are highlighted in red small letters.

Supplementary Table 4: Parasite staging and RNA-scaling factors of collected parasites

Sample:	Approximate staging:	Slide counts [a]:	total RNA yield per 20ml culture:	Number of nuclei per parasite [b]	Scaling factor RNA/nucleus
T5	40 to 5 hpi	93.9% R , 0% T, 6,1% S	5.8 ug	1.83	1.38
T10	2 to 10 hpi	99.6% R, 0% T, 0.4% S	2.3 ug	1	1.00
T15	7 to 15 hpi	100% R, 0% T, 0% S	2.6 ug	1	1.14
T20	12 to 20 hpi	98.0% R, 2% T, 0% S	2.9 ug	1	1.27
T25	17 to 25 hpi	26.2% R, 73.8% T, 0% S	8.9 ug	1	3.90
T30	22 to 30 hpi	4.3% R, 95.2% T, 0.4% S	18 ug	1.39	5.65
T35	27 to 35 hpi	0.9% R, 11.3% T, 87.7% S	64.8 ug	2.69	10.52
T40	32 to 40 hpi	1.7% R, 11.2% T, 87.2% S	149.2 ug	5.42	12.02

[a] Counting as in (Bartfai et al., 2010). R = ring, T = Trophozoite, S = Schizont

[b] Based on values obtained from (Bartfai et al. 2010)
