# Supplementary material for: The nucleosome landscape of Plasmodium falciparum reveals chromatin architecture and dynamics of regulatory sequences
Source: Nucleic Acids Res. 2015 Nov 17;44(5):2110–24. doi: 10.1093/nar/gkv1214 (PMC4797266; doi:10.1093/nar/gkv1214)
Supplement: SUPPLEMENTARY DATA [file supp_gkv1214_nar-02108-h-2015-File008.pdf]

Supplementary Figure 1

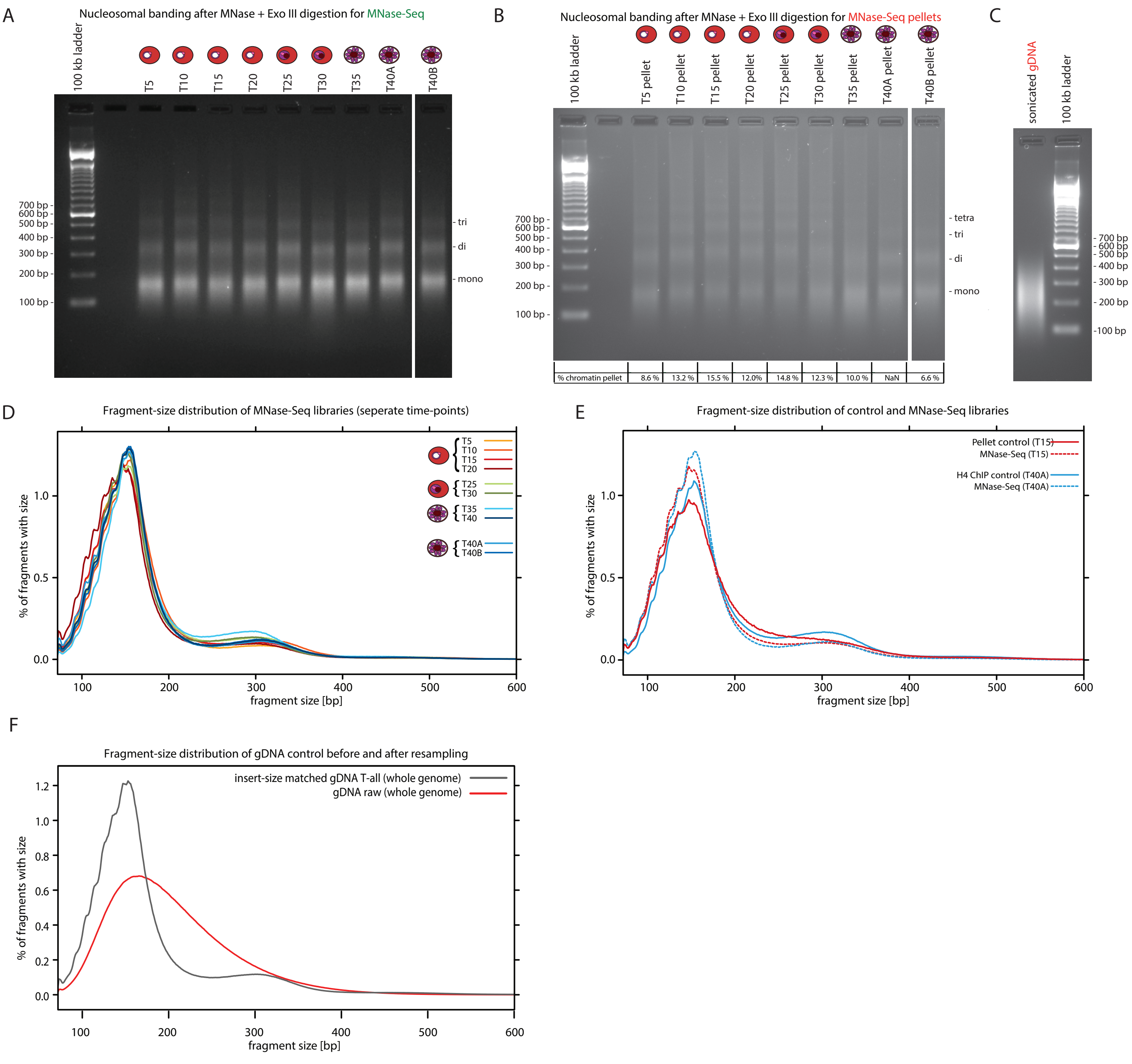

Insert-size distributions & enzymatic digestion of MNase-Seq and control samples to comparable nucleosomal patterns.

(A-C) 2% Agarose gel analysis reveals comparable digestion efficiencies of MNase + exonuclease III-digested chromatin of soluble chromatin (A, MNase-Seq as in Figure 1A) and insoluble pellet-associated chromatin fractions (B, Pellet control as in Figure 1A) from different parasite stages (T5-40). Sheared genomic DNA (gDNA control as in Figure 1A) is fragmented to an extent comparable to chromatin samples, but the distribution is vastly different (C). The proportion of total chromatin remaining associated with the insoluble nuclear membranes is indicated in the table (B, bottom). (D-F) Distribution of fragment sizes inferred from the uniquely aligned read pairs in each MNase-seq library (D), the histone H4-ChIP control sample (T40A, blue) compared to T40A MNase-Seq (blue dashed) and insoluble-nuclear pellet-associated control (T15, red) compared to T15 MNase-Seq (red dashed) (E) as well as in genomic DNA control library before (red line) and after resampling (grey) (F).

Supplementary Figure 2

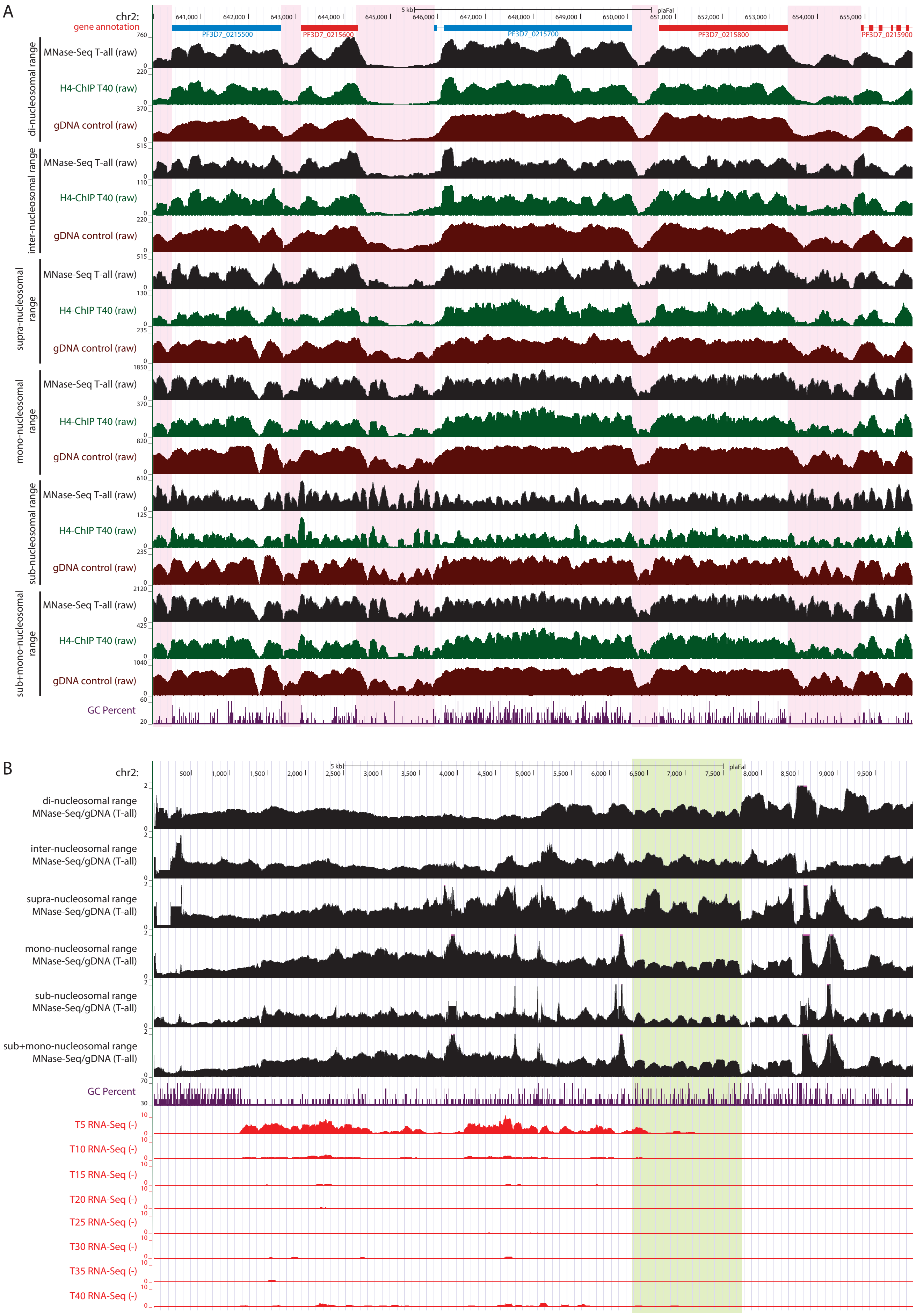

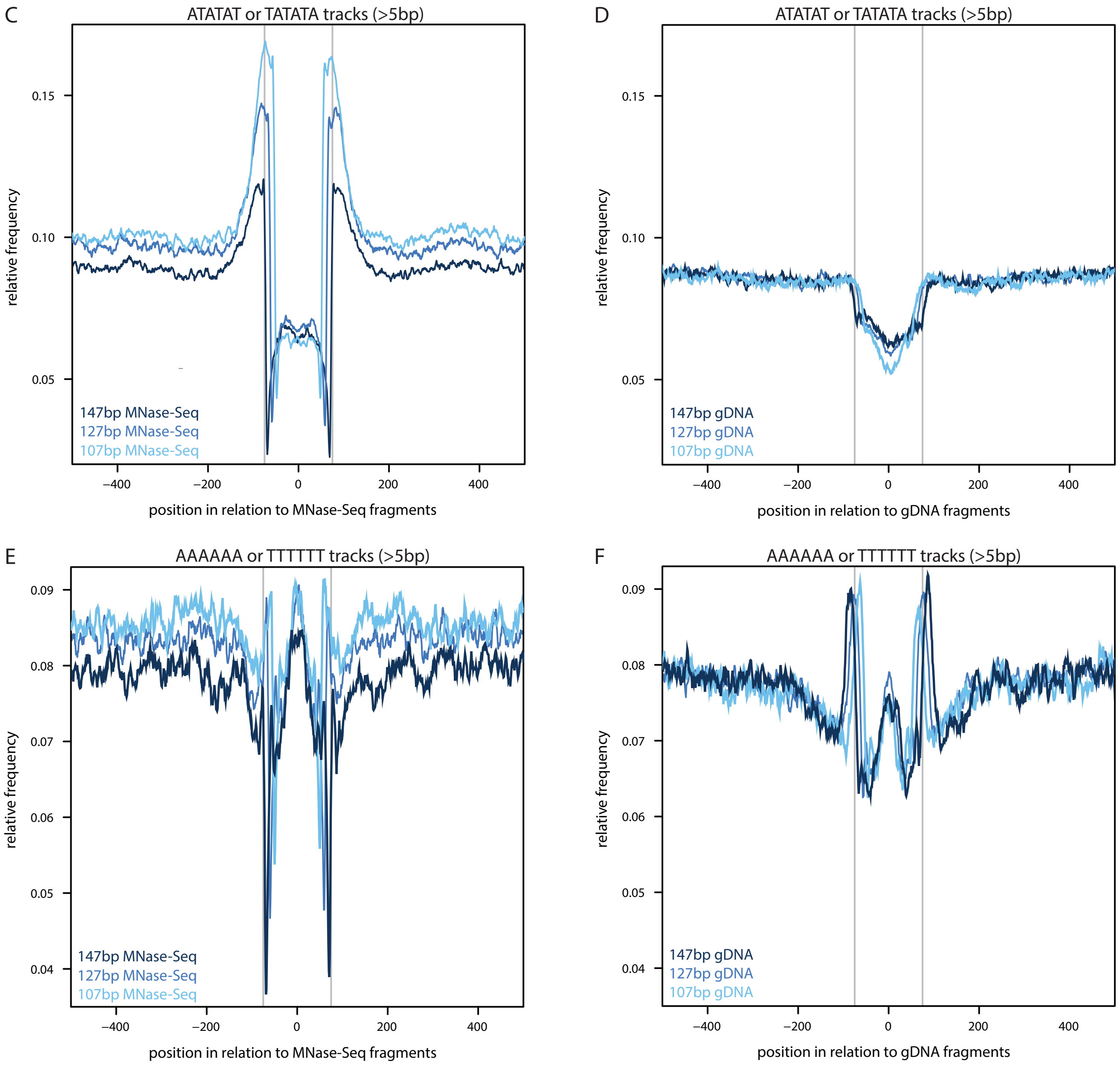

Sub- and mono-nucleosome-sized nucleosome-positioning is evident at intergenic regions and subtelomeric nucleosome-clusters.
(A) Screenshot displaying raw coverage plots of sub+mono-, sub-, mono-, supra-, inter- and di-nucleosomal range of MNase-seq, histone H4-ChIP-seq and matched gDNA control samples. Region depicted is chr2:640,000-656,000. Blue gene: forward strand; Red gene: reverse strand. (B) Screenshot highlighting positioned nucleosome cluster in sub+mono-, sub-, mono- and di-nucleosomal - but not supra- and inter-nucleosomal – size-ranges of MNase-seq fragments corrected by matching gDNA control in the left subtelomere of chromosome 2. Steady-state RNA tracks reveal the presence of a non-coding RNA (red) transcribed towards the telomere. (C-F) Relative frequency of ATATAT/TATATA-type (C-D) or AAAAAA/TTTTTT-type (E-F) repeats in and around all 107, 127 and 147bp MNase-seq (C,E) and gDNA (D,F) fragments. Grey lines border the 147bp size range.

Supplementary Figure 3

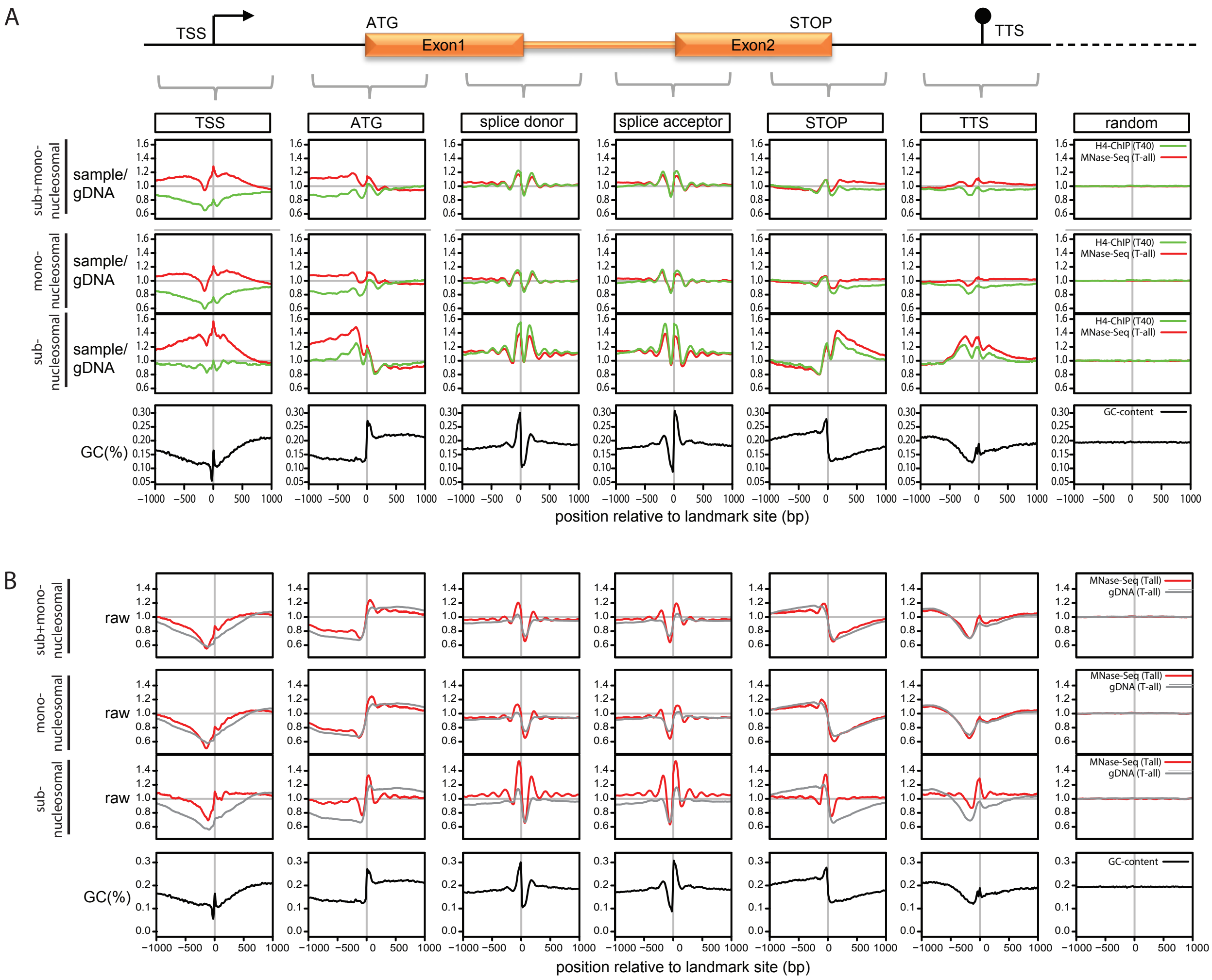

Sub- and mono-nucleosomal size ranges reveal nucleosome positioning, while gDNA-correction is essential to assess nucleosome occupancy

(A-B). Average histone H4-ChIP/gDNA (T40A) and MNase-Seq/gDNA (T-all) (A) or raw MNase-Seq (T-all) and insert-size-matched gDNA (T-all) (B) occupancy profiles around transcriptional landmark sites for sub+mono-, sub- and mono-nucleosomal size ranges. The bottom panels show corresponding GC-content profiles. Ten-thousand non-overlapping random sites were selected uniformly across the genome as control. Raw coverage was normalized by the expected coverage of reads uniformly distributed across the genome.

Supplementary Figure 4

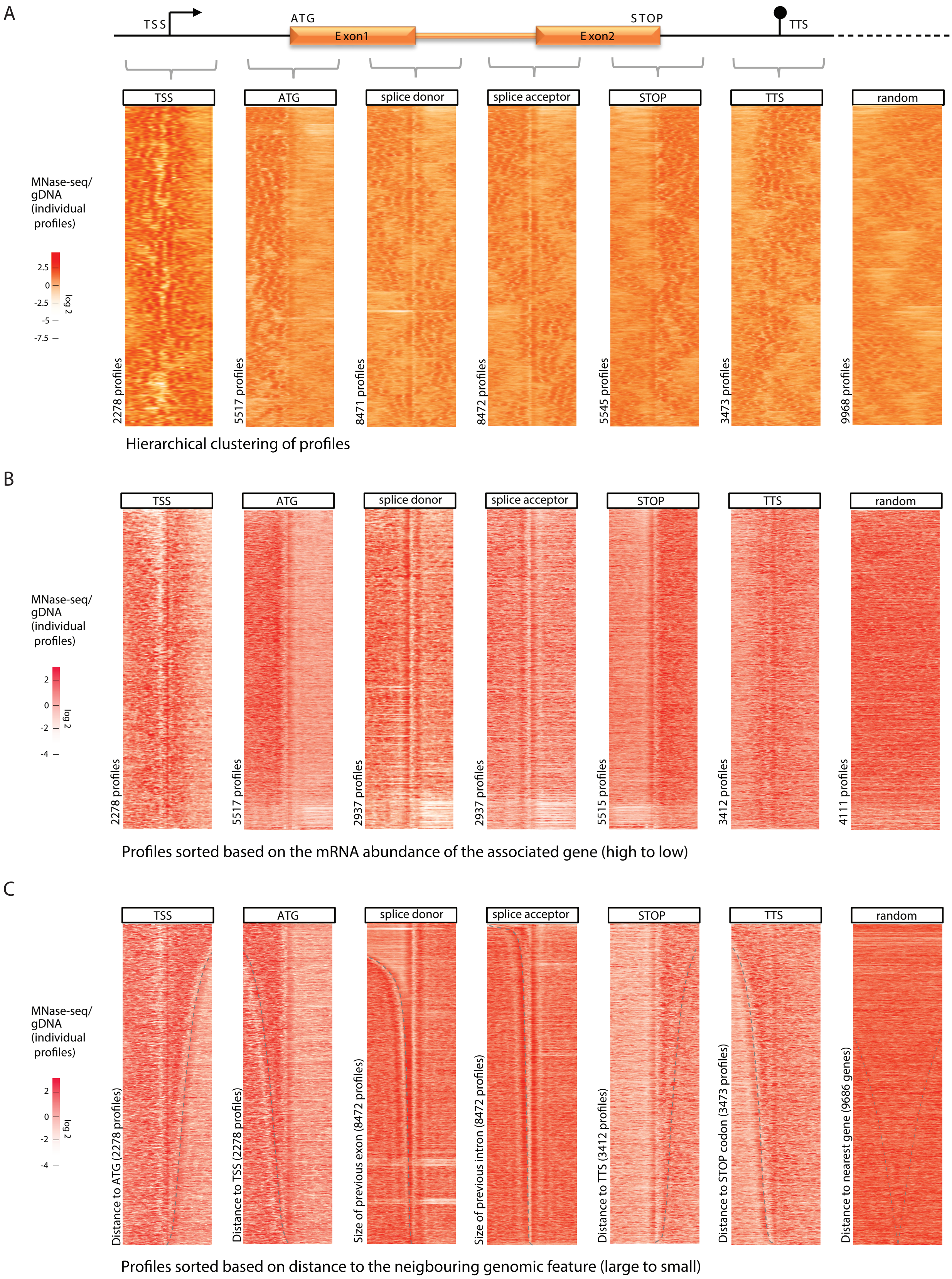

Positioning of nucleosomes on landmark transcriptional sites
Heatmaps depicting individual MNase-seq nucleosome occupancy profiles (T40) +/-1000bp to transcriptional landmark sites normalized by gDNA (log2-MNase-Seq/gDNA ratio). Ten-thousand non-overlapping random sites were selected uniformly across the genome as control. Profiles for all landmark sites were sorted in three different ways: hierarchical clustering (A), mRNA abundance of the corresponding gene (B), distance to the nearest landmark site (C). The number of profiles used is indicated on the side of every heatmaps.

Supplementary Figure 5

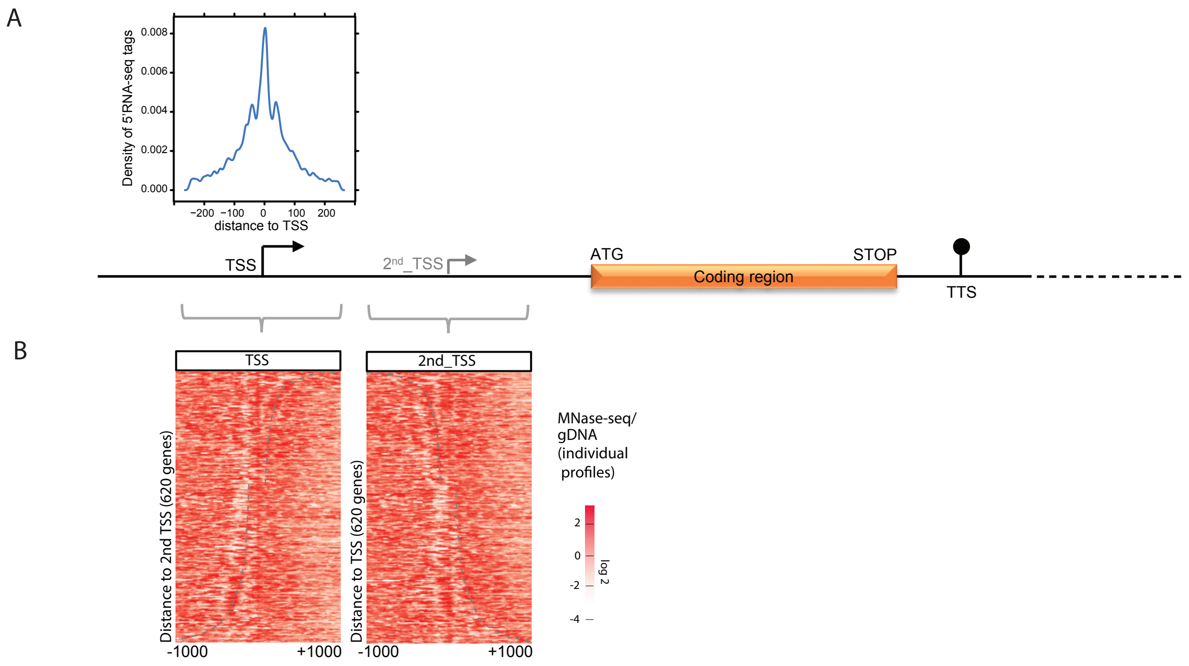

Positioning of nucleosomes in promoters with more than one transcriptional start sites

A) Linegraph depicting the density of 5'RNA-seq tags in relation to the primary TSS.

B) Heatmaps depicting individual MNase-seq nucleosome occupancy profiles (T40) around primary and secondary transcriptional start sites normalized by gDNA (log2-MNase-Seq/gDNA ratio). Profiles were sorted based on the distance to the other TSS, which is indicated by dashed line. Note for this analysis only 2ndTSSs min. 100bp apart from the primary TSS has been considered.

Supplementary Figure 6

A

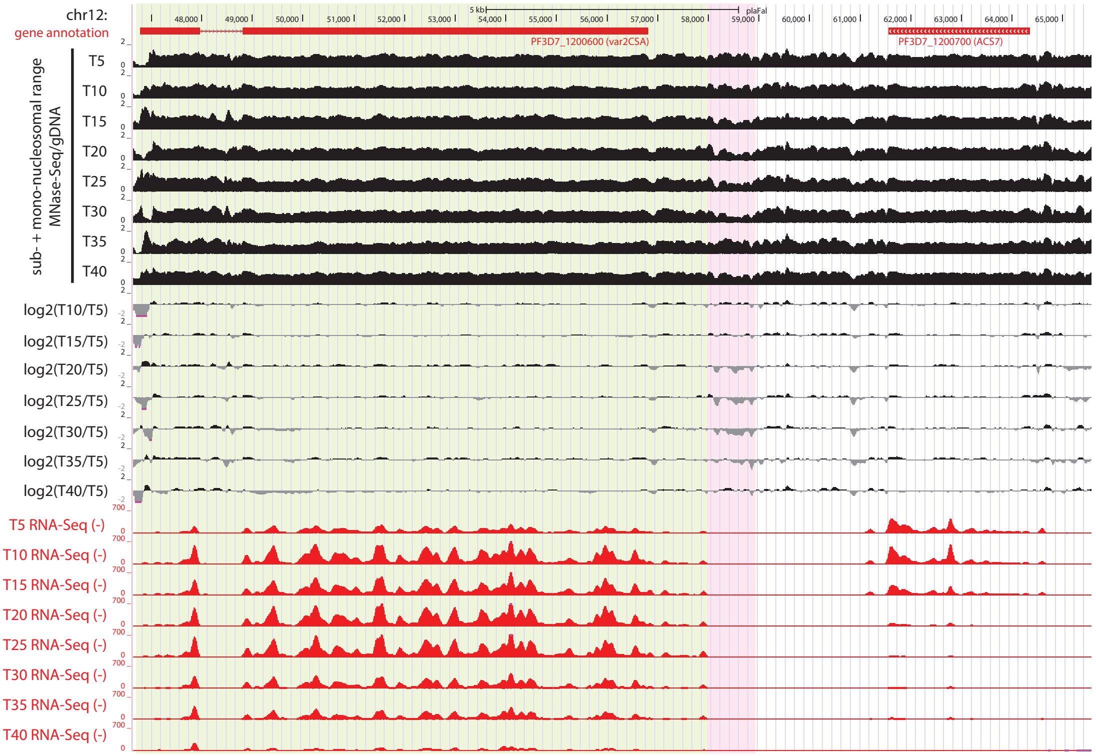

Dynamic nucleosome occupancy in the promoter region of *var2CSA gene*

Screenshot displaying absolute (gDNA-corrected MNase-seq coverage plots) or relative nucleosome occupancy (log2-ratio over T5) and steady-state mRNA expression (strand-specific RNA-seq). Region shown is chr12:46643-65570. Blue gene: forward strand; Red gene: reverse strand; Dynamic regions are highlighted pink, expressed region of *var2CSA* are highlighted green.

Supplementary Figure 7

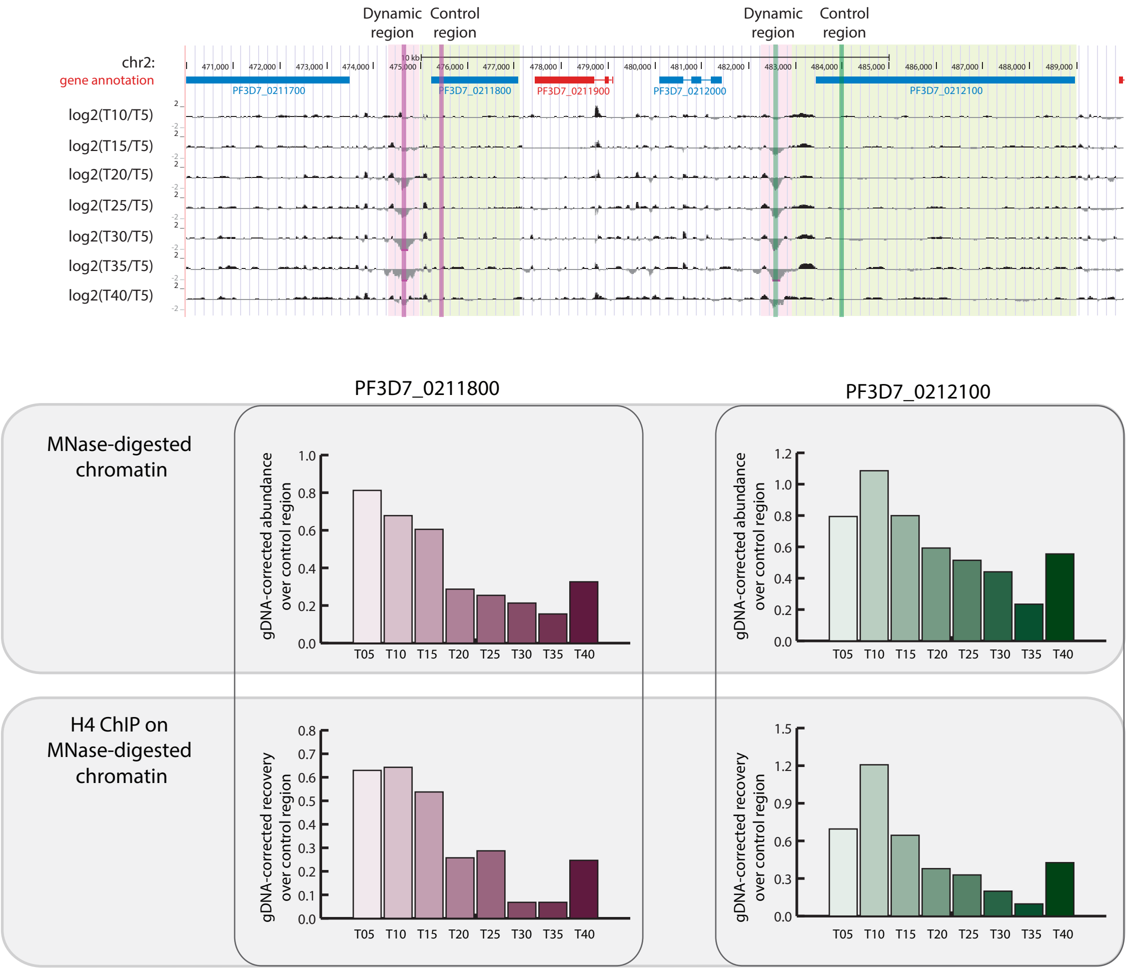

Name	Sequence (5 --> 3')
PF3D7_0221800-dynamic-fw	GCATAAAAAAAAAAAAGAGATG
PF3D7_0221800-dynamic-rev	ATAATATGTTCAATATGTACC
PF3D7_0221800-control-fw	TGTAATGTATTAAATGTTCC
PF3D7_0221800-control-rev	TTGTTTACGTATAGCTTTAC
PF3D7_0212100-dynamic-fw	CAAAGAAAAAATTTAGTTCAC
PF3D7_0212100-dynamic-rev	GGTAGTATTTCCTAATTGC
PF3D7_0212100-control-fw	ACTTCACAAGAAATCAAAAC
PF3D7_0212100-control-rev	GATTCTACATTTTTGTTAGG

qPCR validation of dynamic nucleosome occupancy

qPCRs validation was performed for two genes (PF3D7_0211800, PF3D7_0212100) with dynamic nucleosome occupancy in their promoter regions (screenshot depicts the same region as on Figure 4). For each gene, a primer pair was designed within the dynamic region as well as in a neighboring control region that displayed no change in nucleosome occupancy (highlighted in violet for PF3D7_0211800 or dark green for PF3D7_0212100). Primer sequences are listed at the bottom of the figure.

qPCRs were performed on MNase-digested chromatin before and after α-histone H4 ChIP, respectively. For each primer pairs relative value was calculated in relation to a genomic DNA dilution series. Relative abundance (MNase-digested chromatin) or relative recovery (α-histone H4 ChIP) was calculated by dividing the value for the dynamic region by the value for the respective control region. Importantly, the observed relative occupancy change is primarily the result of the change in the dynamic regions and not that in the control regions (data not shown).

Supplementary Figure 8

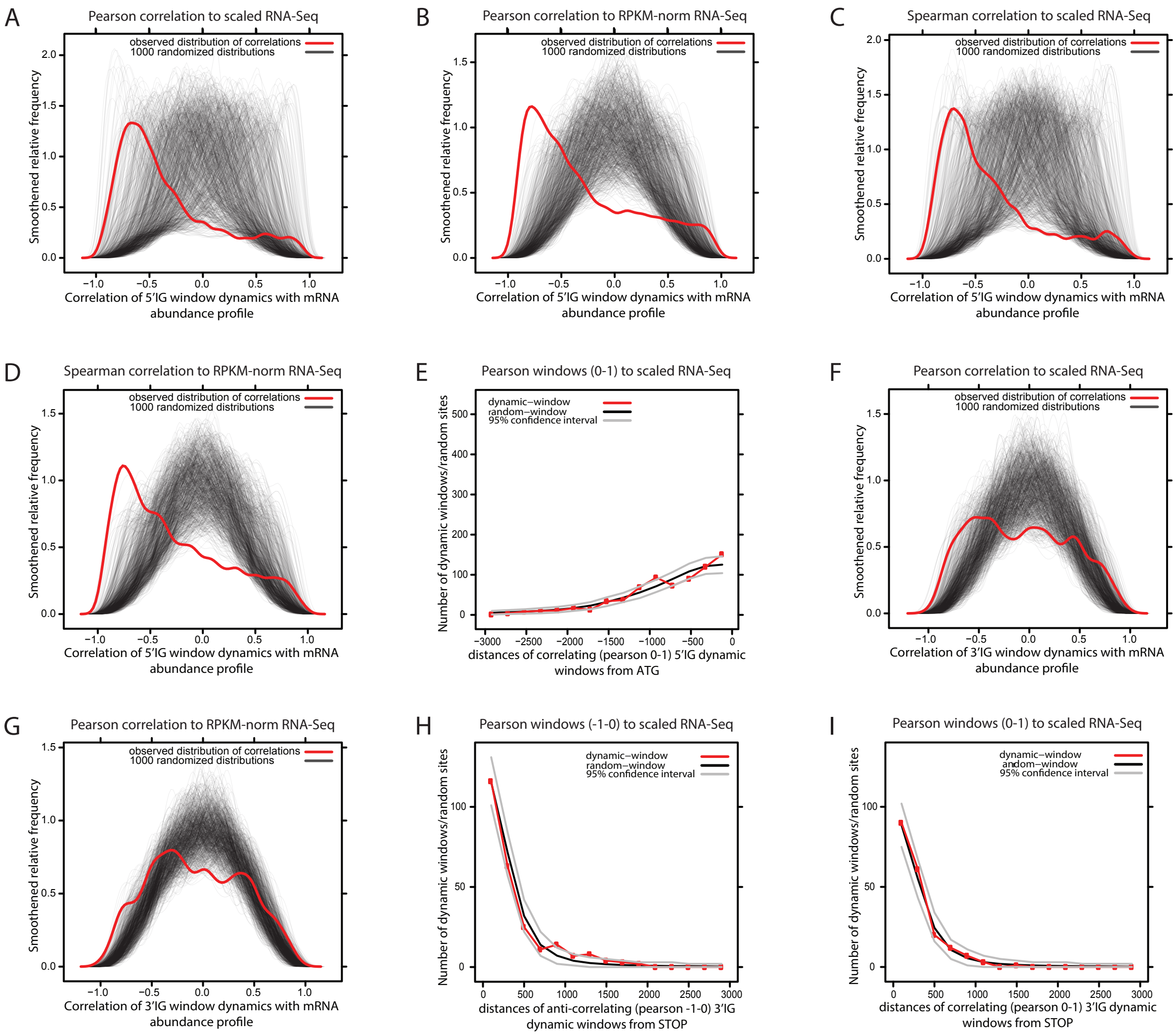

Dynamic nucleosome occupancy versus gene expression and distance to the gene for correlating 5' and (anti)correlating 3' IG regions

(A-D,F-G). Observed (red) and 1000 randomized (transparent black) smoothed distributions (kernel density estimation) of correlations between the nucleosome occupancy of dynamically occupied windows and the expression profile of the cognate gene. Qualitatively similar results are achieved with Pearson (A,B,F,G) and Spearman (C,D) correlation or using RPKM (B,D,G) or scaled RPKM normalization (A,C,F) for both windows located upstream (A-D) or downstream (F-G) of the gene. Every black line shows the distribution of correlations for a single permutation of the time-point labels. (E) Observed (red) and expected (black, grey) distributions of distances between dynamically-occupied upstream windows (with correlation between nucleosome-occupancy and gene expression profile) and ATG of the downstream gene. (H-I) Observed (red) and expected (black, grey) distributions of distances between dynamically-occupied downstream windows (with anti-correlation between nucleosome-occupancy and gene expression profile (H) or correlation between nucleosome-occupancy and gene expression profile (I)) and STOP of the upstream gene. For panels (E,H-I) The mean (black) and 95%-confidence intervals (grey) of the expected distribution were derived from 1000 randomizations of dynamic windows within the upstream/downstream regions. It decays with the distance to the gene as it reflects the number of regions extending to a certain distance (x-axis).
